# Aberrant DNA methylation defines isoform usage in cancer, with functional implications

**DOI:** 10.1371/journal.pcbi.1007095

**Published:** 2019-07-22

**Authors:** Yun-Ching Chen, Laura Elnitski

**Affiliations:** Genomic Functional Analysis Section, National Human Genome Research Institute, National Institutes of Health, Bethesda, Maryland, United States of America; Ottawa University, CANADA

## Abstract

Alternative transcript isoforms are common in tumors and act as potential drivers of cancer. Mechanisms determining altered isoform expression include somatic mutations in splice regulatory sites or altered splicing factors. However, since DNA methylation is known to regulate transcriptional isoform activity in normal cells, we predicted the highly dysregulated patterns of DNA methylation present in cancer also affect isoform activity. We analyzed DNA methylation and RNA-seq isoform data from 18 human cancer types and found frequent correlations specifically within 11 cancer types. Examining the top 25% of variable methylation sites revealed that the location of the methylated CpG site in a gene determined which isoform was used. In addition, the correlated methylation-isoform patterns classified tumors into known subtypes and predicted distinct protein functions between tumor subtypes. Finally, methylation-correlated isoforms were enriched for oncogenes, tumor suppressors, and cancer-related pathways. These findings provide new insights into the functional impact of dysregulated DNA methylation in cancer and highlight the relationship between the epigenome and transcriptome.

## Author summary

In eukaryotes, one gene can be transcribed into multiple RNA sequences (or isoforms) that are subsequently translated into proteins with different functions in response to specific cellular needs. Recent studies showed that cancer cells can obtain abnormal functions via expressing different isoforms. In normal cells, isoform expression can be regulated by DNA methylation–a molecular signature with attached methyl groups on DNA sequences. Given that dysregulation of DNA methylation is a cancer hallmark, we suspect the same regulation holds in cancer and contributes to cancer progression. In this study, we analyzed data from 18 human cancer types and found frequent correlations in 11 cancer types between specific isoform usage and DNA methylation depending on the location of the methylated site in a gene. These correlation patterns can classify heterogeneous tumors in a cancer type into homogeneous subtypes and are predicted to change protein functions via isoform switching between subtypes. Finally, we found cancer-related genes often harbored more DNA methylation-isoform correlations than genes not implicated in cancer. This finding could help us to better understand the functional impact of DNA methylation alterations via regulation of isoform expression in tumorigenesis and to further improve the cancer treatment.

## Introduction

More than 90% of human protein coding genes are capable of producing multiple isoforms, either by adopting alternative transcription start or termination sites (TSSs or TTSs, respectively) or by switching internal splice sites to generate alternative exons [1]. Utilizing these approaches, gene function can be tailored to fulfill specific cellular requirements, including regulating cell-fate in response to stress [2] or nerve cell regeneration after injury [3]. However, isoform switching–differential usage of gene transcripts between conditions–is also common, and often biased in cancer versus normal cells [4], where it is predicted to have deleterious consequences such as sustaining cell proliferation, disturbing apoptosis, and enabling cell motility and invasion [5–7]. Indeed the presence of isoform switching in tumor cells can predict patient survival, independent of cancer type [4]. To date, researchers seeking to learn more about the mechanisms underlying aberrant isoform activity in cancer have primarily focused on mutations in splicing regulatory sites or altered/deregulated splicing factors. This line of study has been fruitful [5, 8, 9]. For example, we now know that mutations in the tumor suppressor gene *BRCA1* cause inappropriate exon skipping and inactivation of *BRCA1* [10], whereas upregulation of *NUMA1* splice isoforms in breast cancer cause increased cell proliferation [11]. However, the effect of highly dysregulated DNA methylation (DNAm), a distinguishing feature of cancer [12], on isoform usage in tumorigenesis has not been fully investigated.

We now know that the methylation of intragenic CpG dinucleotides is known as an important regulatory mechanism for isoform switching in normal cells at promoters, internal splicing sites and transcription termination sites. For example, CpG island (CGI) methylation can regulate the activity of internal promoters to provide tissue-specific activity, as evidenced by differential isoform expression of *SHANK3* in distinct brain regions from a single cell type [13]. Moreover, intragenic DNAm within exons or near exon boundaries can regulate alternative splicing outcomes by (1) preventing access of the DNA-binding protein CTCF, whose presence mediates local RNA polymerase II pausing for inclusion of weak exons or (2) facilitating access of the DNA-binding protein MeCP2, involved in inclusion of alternatively spliced exons [14, 15]. Affecting differential use of transcription termination sites, CGI methylation directs imprinting of murine *H13* isoforms between paternal and maternal alleles [16]. Finally, DNAm also plays a more generalized repressive role by preventing spurious intragenic PolII initiation to ensure transcriptional fidelity throughout gene bodies [17]. Despite abundant evidence that DNAm can regulate isoform switching, research on whether this phenomenon might drive tumorigenesis has been limited, with past studies focusing on alternative transcription start site utilization in prostate cancer [18] or isoform switching among single genes in individual cancer types [19–23].

In this study, recent advances in transcriptome sequencing and DNAm analysis, coupled with expansive collections of tumor samples, enabled us to test the hypothesis that DNAm dysregulation in cancers can disrupt isoform usage and contribute to tumorigenesis, as a common phenomenon. Further, we investigated whether correlated DNAm and isoform disruption explains differences in tumors from the same organ. Based on a comprehensive analysis of data for 18 cancer types from The Cancer Genome Atlas (TCGA), we report that, within 11 cancer types, DNAm in the top 25% of variable methylated sites is associated with isoform switching, and this isoform switching is predicted to have functional consequences for tumorigenesis, involving 10–21% of genes.

## Results

### Correlation between intragenic DNAm and isoform usage in cancer

To investigate the regulatory role of DNAm in isoform production, we first calculated Pearson correlations between intragenic DNAm and isoform usage (defined here as the proportion of a given isoform’s expression over the expression of all isoforms for a gene). We did this for every isoform-methylation probe pair among tumor samples, for all 18 TCGA cancer datasets used in the study (see Methods). A positive correlation indicates that isoform usage increases as DNAm increases, whereas a negative correlation indicates the opposite. We found significant correlations for at least one isoform-methylation probe pair [empirical false discovery rate (eFDR) < 0.1] in 16 out of 18 cancer types examined. Whether or not a cancer type met the significance threshold was mainly governed by sample size: those with fewer tumor samples required higher correlation coefficients, and two were not able to reach the designated eFDR level at all (colon and glioblastoma). The minimal correlation coefficients (measured as absolute values) required to pass the eFDR cutoff ranged from 0.65 in rectum adenocarcinoma ('READ', 33 samples) to 0.16 in breast invasive carcinoma ('BRCA', 268 samples) (S1 Fig). In addition, the significant correlations remained across multiple cancer types using Spearman correlation (S2 Fig), as well as data with batch effects removed (S3 Fig) suggesting the observed significance was not affected by batch effects and outliers when using Pearson correlation.

To further investigate the connection between DNAm and isoform usage, for each cancer type we identified methylation probes with the most variable values, which were most likely to behave differently across samples (i.e., those whose standard deviation across tumors fell within the top 25% for all sites). From these highly variable methylation sites, we required an absolute correlation between DNAm and isoform usage > 0.3. To guarantee all of these pairs are significantly correlated, we excluded seven cancer types whose absolute correlation was above 0.3 at FDR = 0.1. Although this cutoff value lowered the number of cancer types, it yielded a substantial number of correlated isoform-probe pairs for downstream analyses in each of the 11 remaining cancer types (see 11 cancer types and their abbreviations in Table 1). Depending on the cancer type, we identified significantly correlated isoform-probe pairs from 10% (n = 1,428 in thyroid carcinoma, or 'THCA') to 21% (n = 3,121 in bladder urothelial carcinoma, or 'BLCA') of genes analyzed. We hypothesized that, using this dataset, we could determine whether the location of DNAm in an isoform affects its usage; thus, these data form the basis for the analyses that follow.

**Table 1:** The number of tumor samples, normal samples, genes, transcript isoforms, and isoform-probe pairs being analyzed across 11 cancer types.

Cancer Type	Abbr.	# Tumor	# Normal	All data analyzed			|correlation| > 0.3 (top 25% variable probes)		
				# Gene	# Trans	# Pair	# Gene	# Trans	# Pair
bladder urothelial carcinoma	BLCA	161	4	14542	82868	1644048	3121 (21%)	6286	18285
breast invasive carcinoma	BRCA	268	33	15101	89913	1794567	1770 (12%)	3233	8992
head and neck squamous cell carcinoma	HNSC	201	7	14610	84086	1678216	1666 (11%)	2985	8468
liver hepatocellular carcinoma	LIHC	136	19	13580	70564	1378646	2030 (15%)	3653	11898
lung adenocarcinoma	LUAD	177	6	15151	90076	1796652	1844 (12%)	3288	9507
lung squamous cell carcinoma	LUSC	161	2	15299	92539	1846398	2549 (17%)	5007	15141
prostate adenocarcinoma	PRAD	189	13	15003	85718	1723875	1991 (13%)	3570	10992
skin cutaneous melanoma	SKCM	175	0	14455	84608	1695090	2482 (17%)	5315	17449
stomach adenocarcinoma	STAD	147	0	15387	97792	1974208	2345 (15%)	4338	11718
thyroid carcinoma	THCA	191	20	14863	87044	1739557	1428 (10%)	2575	5399
uterine corpus endometrial carcinoma	UCEC	169	13	14340	76168	1513015	1533 (11%)	2648	8652

Trans = transcript isoform

### DNAm around the transcription start site is correlated with decreased isoform usage, irrespective of CpG island presence

**Fig 1:**
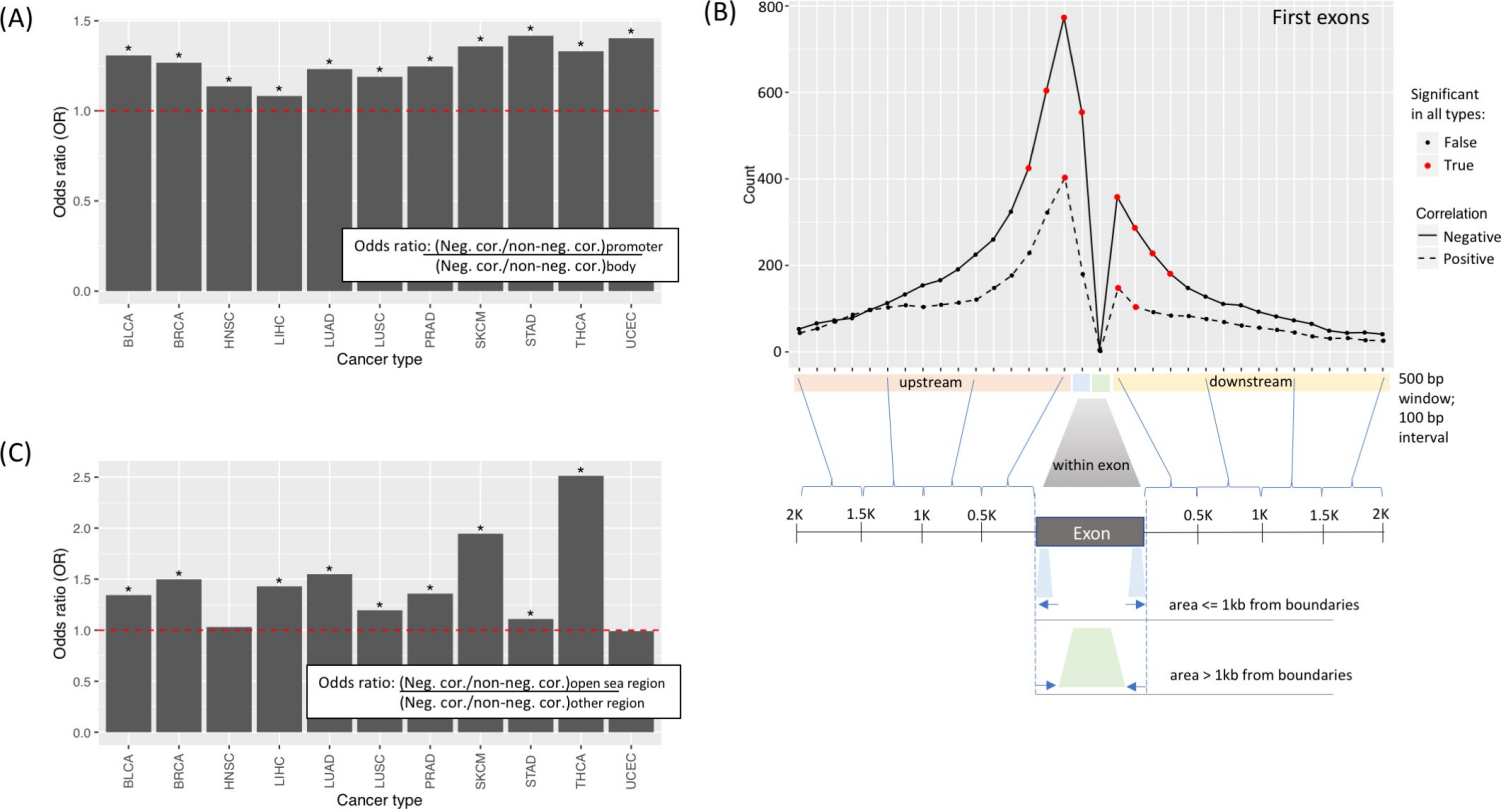
Negative correlations between isoform usage and DNAm show enrichment in promoter regions relative to isoform bodies. (A) The odds ratios were computed between the ratio of negative versus positive correlations in isoform promoters and isoform bodies for each cancer type. (B) The median count (among 11 cancer types) of positive and negative correlations computed in binned regions of 500 bp. Regions include upstream, within, and downstream of first exons. Each dot represents a sliding window of 500bp with a sliding interval of 100bp. Significant or nonsignificant windows in 11 cancer types are indicated by red and black dots. Negative and positive correlations are shown with solid and dashed lines. Distances within exons of < = 1kb or >1kb from exon boundaries are illustrated. (C) Negative correlations within 1kb around the first exon boundaries were enriched in open sea regions compared to CGIs or shores and shelves (OR>1).

For each cancer type, when DNAm was found in isoform promoters (defined as the areas upstream of the TSSs or downstream, including the first exon), negative correlations between DNAm and use of that isoform were overrepresented (see Methods, Fig 1A). This indicates DNAm at the TSSs acts as a negative regulator of isoform usage. This type of regulation was supported by a median number of 924 genes across 11 cancer types (denoted as #gene = 924). To further investigate rules governing DNAm and TSS usage, we investigated all DNAm probe sites that fell within isoforms' first exons and flanking regions (including 2-kb upstream noncoding regions and 2-kb downstream intronic regions). Across all cancer types, we found that within the 1-kb regions upstream and downstream the first exon boundaries (including inside the first exon), negative correlations were enriched between DNAm and isoform usage (#gene = 753) (Fig 1B). Despite the dominance of negative correlations, some positive correlations were enriched across all cancer types between DNAm and isoform usage among probes within 500 bp of the first exon (#gene = 232), suggesting that some promoter methylation may associate with transcriptional activation.

Within the set of negatively correlated sites located within 1-kb of the first exon boundaries, we tested whether the methylation sites were located in CGIs–a well-known cause of transcriptional silencing in cancer. To do so, we examined probes for overrepresentation in CGIs, shores and shelves (SSs) or open sea regions (OSRs); (i.e., SS = the 4-kb regions flanking CGIs, and OSRs = areas that are not CGIs or SSs). We found negative correlations enriched among probes within OSRs, compared to other regions, in 9 out of 11 cancer types (#gene = 232) (Fig 1C) whereas the same enrichment was seen for CGIs in only 6 out of 11 cancer types, suggesting that TSS-correlated DNAm need not be confined to CGIs to impact transcription initiation.

### DNAm in downstream isoform positions is correlated with increased isoform usage

**Fig 2:**
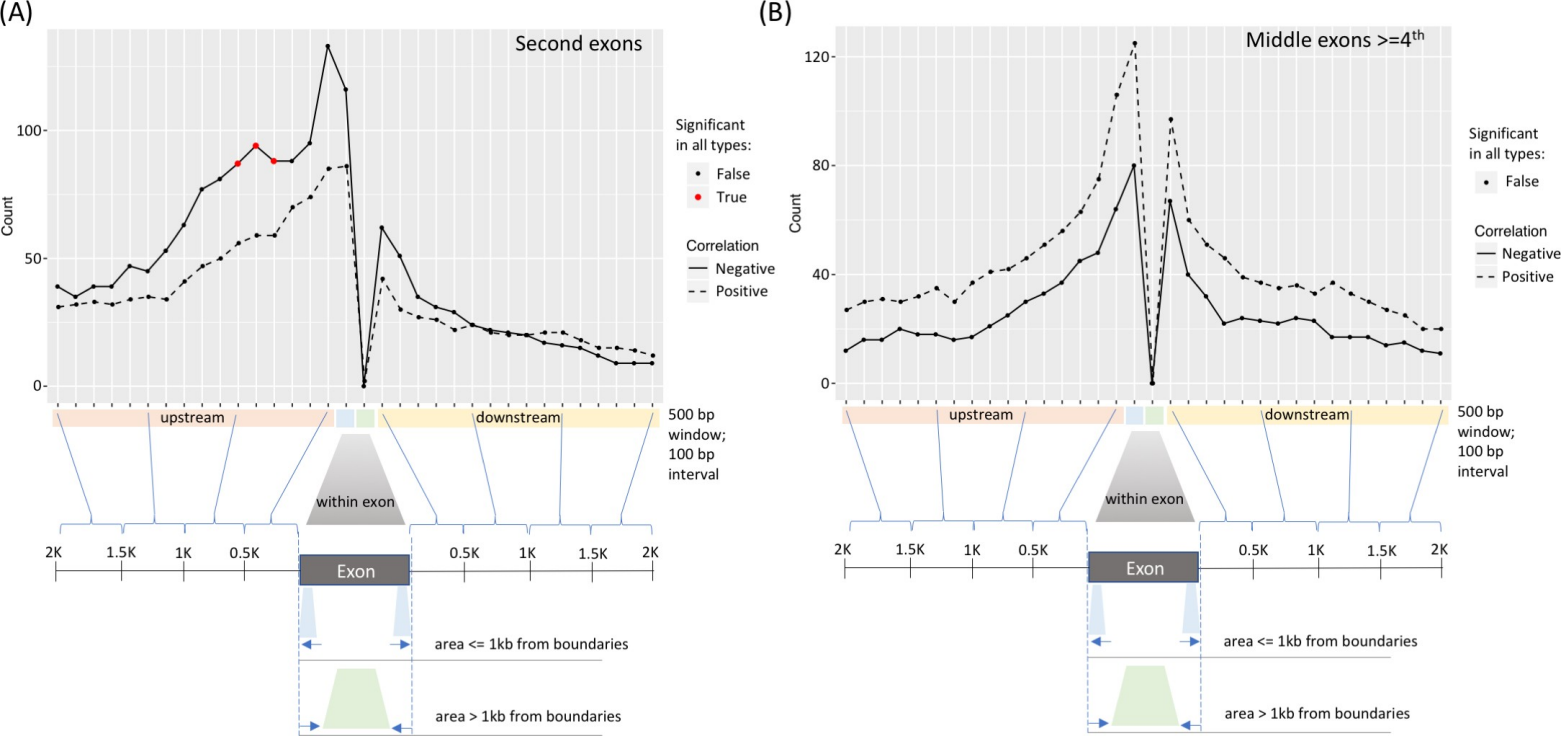
Negative correlations occur upstream of second exons, whereas positive correlations occur at the fourth position or later. (A) The median count (among 11 cancer types) of positive and negative correlations computed for each binned region inside and around the middle exons in the second position in the exon-intron isoform structure. Negative correlations in the upstream were interpreted as TSS-related repression. (B) The same plot as in (A) but for middle exons in the fourth or later position, showing majority of positive correlations. Significance across all cancer types was not detected in the fourth exon (or later) data.

Next, we investigated correlations between DNAm within the isoform body (defined as the areas downstream of first exons) and isoform usage. Here, positive correlations were overrepresented (#gene = 888; S4 Fig). This suggests that DNAm in isoform bodies plays a role in transcriptional elongation. To take a closer look, we confined our analysis to methylation probes around isoforms' middle exons, defined as exons that were neither the first exons (i.e., those containing TSSs) nor the terminal exons (i.e., those containing TTSs). Around the second exon, we unexpectedly found enrichment of negative, rather than positive correlations between DNAm and isoform usage extending from 300 bp to 1 kb upstream (#gene = 84) (Fig 2A). We reasoned this enrichment could be due to repression of transcription at the first exon, given that the median distance between first and second exons was minimal (i.e., 266 to 795 bps depending on cancer type). Around the third exon, no enrichment in either positive or negative correlations was observed. However, flanking fourth exons and beyond, more positive than negative correlations occurred (Fig 2B). These findings suggest that, across most cancer types, DNAm at isoform positions > = exon 4 correlates with inclusion of distal exons in the gene body, indicative of transcriptional elongation.

### DNAm near the TTS may define the 3´ isoform boundary

**Fig 3:**
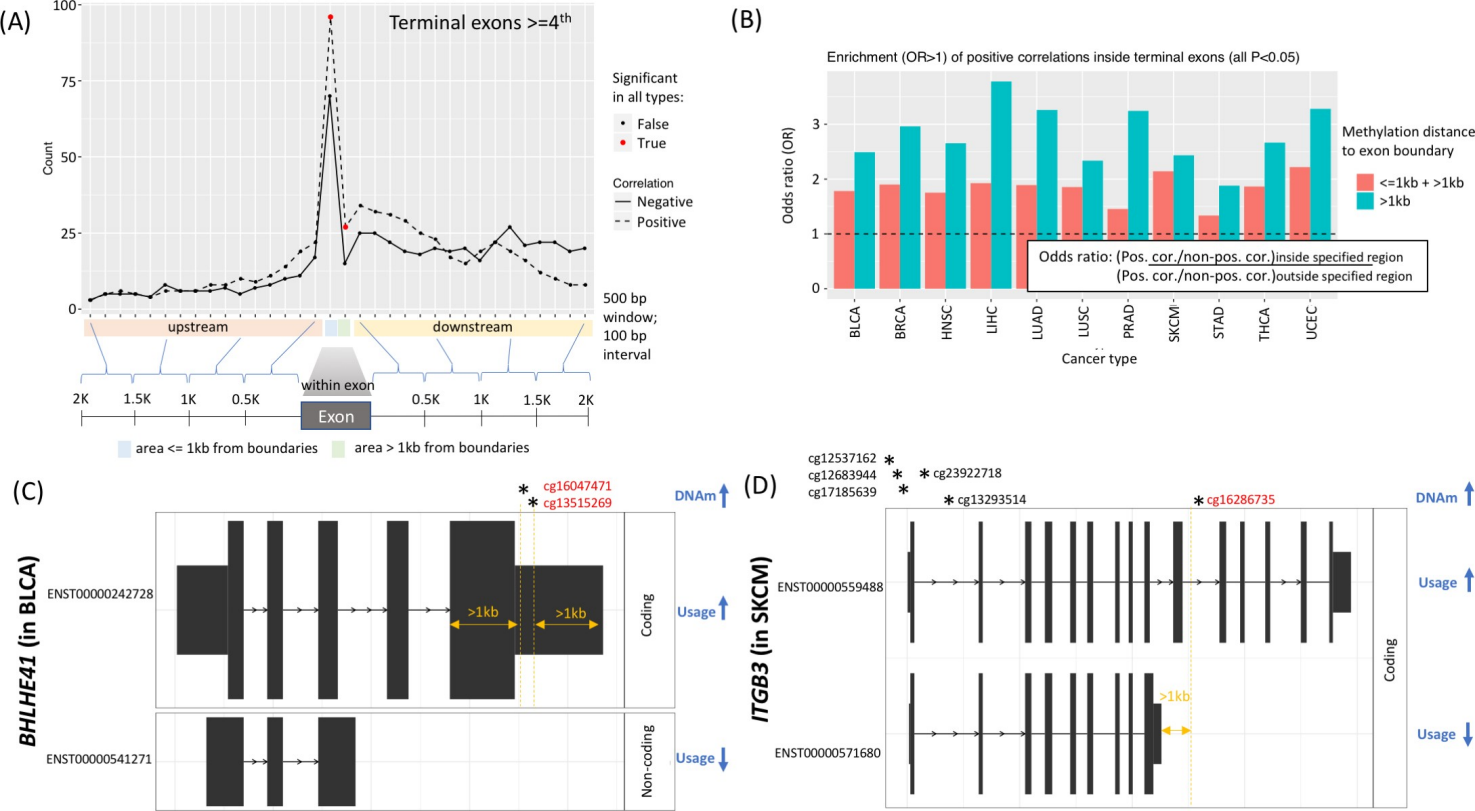
DNAm positioned within the terminal exon or relative to an alternative, downstream terminal exon correlates with inclusion. (A) Median count (across 11 cancer types) of positive and negative correlations between DNA methylation and isoform use was computed for each binned region inside and around the terminal exons in the fourth exon position or beyond (see schematic below plot). Significance across all cancer types is seen only when methylation appears within the terminal exon (red circles). (B) Positive correlations between DNA methylation and isoform use are enriched when DNAm is within terminal exons, both > and < 1 kb from the exon boundaries (OR>1), especially in long exons where boundaries are more than 2 kb away (blue bars). (C) In bladder cancer (BLCA), DNAm within the exon and far from either exon boundary (>1kb) in *BHLHE41* is correlated with the inclusion of the long terminal exon, resulting in increased usage of the longer isoform but decreased usage of the shorter one. (D) In melanoma (SKCM), DNAm in *ITGB3* that lies downstream of and ≥ 1 kb away from the terminal exon of the shorter isoform is positively correlated with usage of the downstream TTS located in the longer isoform, but negatively correlated with the shorter isoform. Five CpG sites (asterisks) near the transcription start site are also positively correlated with the longer isoform but negatively correlated with the shorter one.

When we repeated the same analysis for probes around the terminal exons to examine DNAm and use of terminal exons (i.e., exons containing TTSs that were not first exons), we found enrichment in positive correlations between DNAm and isoform usage only for probes in or around terminal exons that occupied the fourth or later exon positions (Fig 3A). This trend was not observed for terminal exons that occupied the second or third exon positions.

For terminal exons in the fourth exon position or beyond, we found an enrichment of positive correlations (p<0.05) across all cancer types for DNAm probes located within those exons (#gene = 73) (Fig 3A). When we pooled all terminal exons regardless of their positions within the isoform (2^nd^ + 3^rd^ + 4^th^ + later; #gene = 125), we found evidence for slightly more positive correlations and enrichment remained for all cancer types. The signal was strongest when we focused on those DNAm probes whose distance to either boundary of the terminal exon was >1kb (#gene = 20), indicating exon length greater than 2 kb (Fig 3B). Furthermore, these positively correlated methylation probes were enriched in CGIs (#gene = 6) (S5 Fig). Collectively, these findings suggest that DNAm in terminal exons may be a signal for terminal exon inclusion, where the signal intensifies at long exons with CGI methylation.

We also examined DNAm existing outside the terminal exons and asked whether correlations gave insight to TTS use. We found more positive DNAm correlations than negative correlations, up to 1 kb downstream of the terminal exon but not beyond this distance (Fig 3A). Thus, we tested the hypothesis that DNAm in distal downstream locations of a gene may correspond to usage of a latter TTS rather than a former TTS when alternative TTSs are present. Across all 11 cancer types, we collected 10,467 examples of DNAm-correlated isoform switching in which methylation at a single DNAm probe was positively correlated with use of one isoform of a gene but negatively correlated with use of another (this number excluded examples of DNAm-correlated alternative TSSs). Of these, we assessed the distance from the DNAm site to the correlated terminal exon and identified 5,707 instances in which the DNAm probe was located 1 kb beyond the preceding terminal exon boundary. In 70% of those cases (3,977/5,707), the DNAm site was negatively correlated with the use of the preceding TTS, but positively correlated with use of the more downstream TTS. These data support a hypothesis that in cases of isoform switching, DNAm more than 1 kb beyond a terminal exon can be a marker for use of an alternative, more distal TTSs.

Our findings are illustrated by two (DNAm-correlated) isoform switches we detected in tumor suppressor genes: *BLHLE41* (in BLCA) and *ITGB3* (in skin cutaneous melanoma, or 'SKCM'). In both cases, the DNAm corresponded to RNA isoform output (Fig 3C and 3D). For example, the very long terminal exon in *BHLHE41* contains two internal methylation sites positively correlated with the inclusion of a long terminal exon but negatively correlated with the shorter isoform (Fig 3C). The shorter isoform is noncoding and lacks the helix-loop-helix and 'hairy_orange' domains, known for DNA binding and interaction with repressive chromatin modifying enzymes (S6A Fig). Similarly, in *ITGB3*, methylation of a site located >1 kb beyond the terminal exon of the shorter isoform positively correlates with use of the longer isoform and alternative TTS but negatively correlates with the shorter isoform (Fig 3D). Here, both isoforms produce coding transcripts, but the shorter one lacks EGF_2 domains and integrin tail and cytoplasmic domains, necessary for activation of Src tyrosine kinase signaling used in cell movement and proliferation (S6B Fig). Of note, we identified DNAm at five sites near the TSSs also positively correlated with the longer isoform, but negatively correlated with the shorter one, suggestive of additional epigenetic relationships in isoform regulation.

### Correlated DNAm and isoform usage can be used to classify tumors into known subtypes

In previous studies, DNAm patterns have been used to classify tumors into known or novel subtypes, with the goal of reducing heterogeneity and gaining insight into molecular similarities via unsupervised hierarchical clustering. We investigated whether DNAm patterns corresponding to distinct gene isoform usage could also delineate known tumor subtypes. To address this question, we performed unsupervised hierarchical clustering on samples using DNAm data from sites whose methylation was correlated with isoform usage for each of the 11 cancer types.

**Fig 4:**
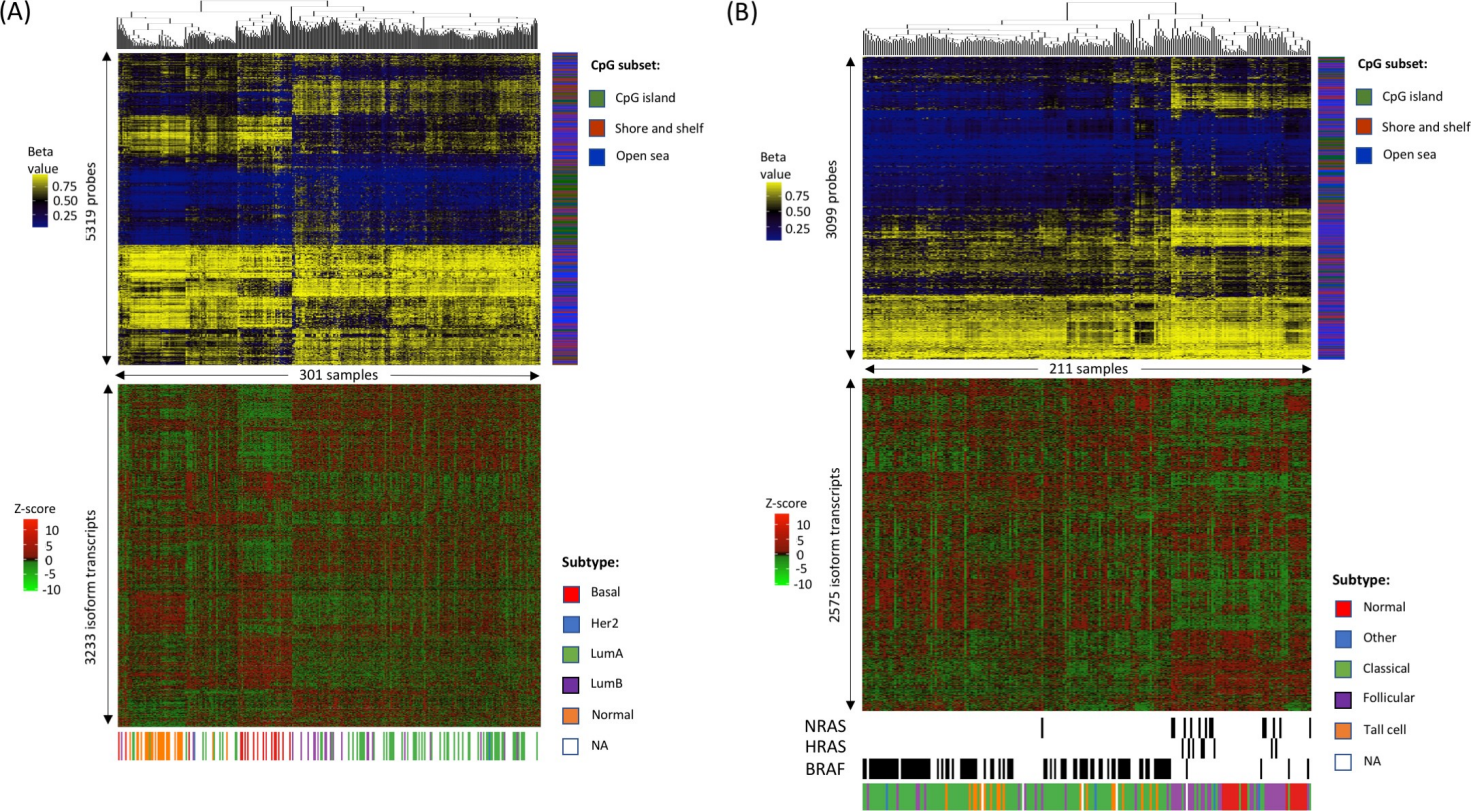
Clustering patterns of correlated DNA methylation and isoform usage patterns are consistent with predefined cancer subtypes. Two vertically aligned heat maps were generated using correlated DNAm patterns (*top*) and isoform usage patterns (*bottom*) for (A) breast cancer and (B) thyroid cancer. Columns in the methylation heat maps represent samples clustered based on DNAm levels of isoform-correlated probes. Columns in the isoform usage heat maps represent usage levels of DNAm-correlated isoforms of clustered samples. Abbreviations: Her2, Her2-positive; LumA, luminal A; LumB, luminal B. Location of DNAm probes as a subset of CpG positions (CGI, shores and shelves or open sea regions) are shown in side bar. Driver mutations are shown for thyroid cancer samples (*NRAS*, *HRAS*, *BRAF*).

BRCA clusters recapitulated previously defined molecular subtypes (Fig 4). The majority of basal and luminal A breast tumors, as well as normal tissue samples, clustered according to their molecular subtypes, displaying distinct DNAm patterns and correlated isoform usage patterns (Fig 4A). Luminal B and luminal A samples were largely intermixed, but they formed a cluster distinct from basal and normal samples. Samples without any subtype annotations (*white bars*) clustered with samples having defined subtypes, indicative of similar methylation and isoform use patterns, which may identify their primary subtypes.

Similarly, thyroid carcinoma 'THCA' clusters recapitulated previously defined histological subtypes. Classical and follicular histological subtypes formed distinct clusters differentiated by both DNAm and correlated isoform usage patterns (Fig 4B). Moreover, these clusters corresponded to molecular subtypes defined by somatic mutations in *BRAF/HRAS/NRAS*. Although classical and tall-cell tumor samples clustered with one another, they remained separated from follicular tumor and normal tissue samples.

In the nine remaining cancer types, we found several examples in which DNAm and isoform usage clustering patterns were upheld by subtype designations (S7 to S15 Figs). For example, in BLCA, head and neck squamous cell carcinoma (HNSC), prostate adenocarcinoma (PRAD), and SKCM, clusters displayed distinct DNAm and isoform usage patterns consistent with many subtypes predefined in TCGA analyses. This suggests that DNA methylation and related isoform usage are tightly coordinated in many cancer subtypes. In liver hepatocellular carcinoma (LIHC), lung adenocarcinoma (LUAD), lung squamous cell carcinoma (LUSC), stomach adenocarcinoma (STAD) and uterine corpus endometrial carcinoma (UCEC), although DNAm clusters were significantly associated with their correlated isoform usage patterns, DNAm clusters were stronger visually, suggesting that the expression changes are small in magnitude.

### Functional implications of DNAm-correlated isoform switching in cancer

To determine whether these subtype-discerning, isoform switching-linked DNAm alterations could impact tumorigenesis, we analyzed the functional outcomes of isoform switching among BRCA subtype samples, and also in normal breast tissue samples, using software designed for this purpose, IsoformSwitchAnalyzeR [4]. Collectively, the isoform switches were predicted to cause functional protein changes in six possible ways: (*i*) by modifying coding potential; (*ii*) swapping functional domains; (*iii*) causing gain or loss of introns; (*iv*) inducing nonsense-mediated decay; (*v*) changing the length of open reading frames; or (*vi*) toggling signal peptide inclusion, which is important for protein secretion. Next, we tallied the number of affected genes for each of these categories of predicted functional change in pairwise comparisons of basal, luminal A, luminal B, and normal samples (S16 Fig). For each comparison except luminal A vs luminal B, we found more than 20 genes affected by one or more type of predicted functional change. Most functional changes involved a protein domain gain or loss, a switch from a coding isoform to a noncoding isoform or vice versa, or extension or shortening of an open reading frame. For example, 42 genes were predicted to lose functional domains after a switch from the isoform common in normal samples to the isoform common in luminal B samples (S16 Fig). These findings suggest that DNAm-correlated isoform switching can alter gene functions, providing a mechanistic explanation for the molecular/histological differences between tumor subtypes and between tumor and normal samples.

**Fig 5:**
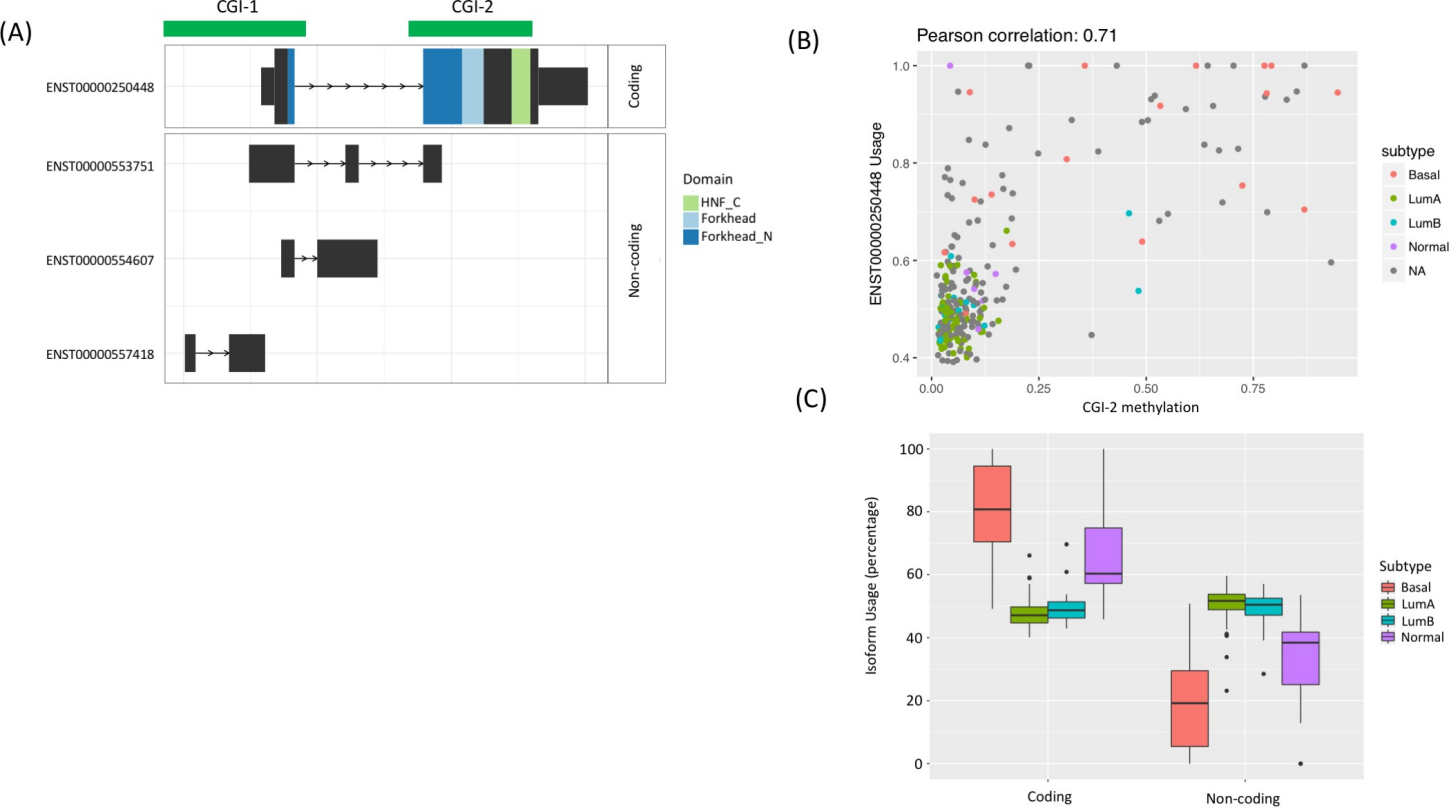
In breast cancer samples, DNAm at *FOXA1* is correlated with subtype-linked switching from coding to non-coding isoforms. (A) A schematic plot of the *FOXA1* locus shows two CpG islands (CGIs), a coding isoform (*top*, with functional domains labeled), and three non-coding isoforms *(bottom*). CGIs shown in dark green. (B) Across the breast cancer subtypes and normal samples, DNAm in CGI-2 of the coding *FOXA1* isoform (ENST00000250448) was positively correlated with differential (coding) isoform usage. (C) Box plots indicate significant differences (P < 0.05; Wilcoxon rank sum test) in usage of the *FOXA1* coding isoform and non-coding isoforms between basal vs luminal A and B samples, and between basal subtype and normal samples.

To take a closer look at one such alteration, we examined *FOXA1*, whose expression is associated with extended disease-free survival in BRCA [24]. Expression of the long terminal exon was correlated with DNA methylation levels (r>0.7) (Fig 5A and 5B). We found evidence for switching between the coding and noncoding isoforms that was relevant to tumor subtypes, where higher usage of the coding isoform occurred in basal tumors and normal samples, and higher usage of the noncoding isoforms occurred in luminal A and B tumor types (Fig 5C).

### DNAm-isoform correlations denote genes enriched in cancer and other biological pathways

We predicted that if DNAm-correlated isoform alterations play an important role in cancer, genes relevant to tumorigenesis should be affected more often than genes that are not. To test this hypothesis, we first identified 53 genes that met our conditions for DNAm-isoform usage correlations across all 11 analyzed cancer types (where |r| > 0.3). Of these genes, nine (16%) were annotated as oncogenes or tumor suppressor genes by Cosmic [25] and the TSGene database [26] (hypergeometric test; p = 7E-4), yielding statistical significance for enrichment as well as evidence of importance in cancer phenotypes. Next, we expanded the pool of genes by varying the least number of cancer types across which correlations were shared (*n*). As the number of cancer types decreased, more genes were recovered, causing enrichment for oncogenes and tumor suppressor genes to decrease from 12% (at *n* = 10; 19 genes) to 8% (at *n* = 2; 374 genes). Nevertheless, the overrepresentation remained statistically significant across all *n* values (hypergeometric test; p < 1E-2) (S1 Table), suggesting DNAm-correlated isoform alterations may be positively selected in cancer-related genes, consistent with our hypothesis.

To understand whether molecular pathways important to carcinogenesis were disproportionately affected by the isoform switching we observed, we selected a set of 1,222 genes that showed correlations between DNAm and isoform usage across more than half of the 11 cancer types (*n* ≥ 6). Pathway enrichment analyses identified 479 pathways (or gene sets) from 7 databases that were significantly enriched (q<0.05), involving 637 of the 1,222 genes (S2 Table). Top pathways included the cytoskeleton, focal adhesion, actin binding, Ras/GTPase signaling transduction, SH3/PH/RhoGEF protein domains, developmental biology, and cancer. This suggests that DNAm-correlated isoform alterations in cancer can be common in genes that promote cancer formation, growth, and metastasis.

**Fig 6:**
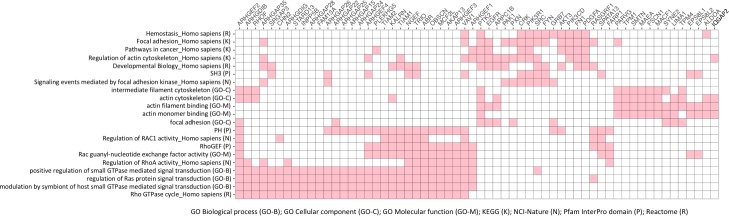
Genes exhibiting DNAm-correlated isoform usage in multiple cancer types show pathway and functional relationships. Red squares indicate involvement in 21 pathways/gene sets (*rows*) for 60 genes with DNAm-correlated isoform usage (*columns*). The 21 pathways/gene sets are the most frequently enriched from assessments of seven pathway/gene set databases (*listed on bottom left*). The genes were part of a larger set of 1,222 genes whose DNAm and isoform usage were correlated in at least 6 (out of 11) cancer types.

Given the frequent gain/loss of functional domains in DNAm-correlated isoform switching, we wondered whether genes involved in the top pathways identified above encoded particular protein domains whose loss could affect their functional roles. We visualized the genes by performing hierarchical clustering on 60 out of the 1222 genes that were most frequently involved in 21 top pathways or gene sets. We found clustering of numerous genes with PH or RhoGEF domains that were assigned to GTPase/Ras signal transduction, and a number of distinct genes with SH3 domains, which were assigned to developmental biology and cancer pathways (Fig 6). Thus, alteration of the DNAm of these genes may correspond to alteration of their functional domains and influence pathway functions in many types of cancer.

## Discussion

In this study, we showed that intragenic DNAm is correlated with isoform usage across numerous cancer types, tying together the aberrantly modified epigenome and the transcriptome. Within cancer types, DNAm correlated to isoform usage patterns can be used to classify tumors into known histopathological subtypes, implicating distinctive protein alterations in functional differences among tumors. Furthermore, we show that DNAm-correlated isoform usage is enriched in oncogenes and tumor suppressor genes as well as pathways pertinent to tumorigenesis, such as cell adhesion and signaling. Thus, this study shows that DNAm-correlated isoform usage alterations are common, could functionally contribute to cancer processes, and represent a new paradigm in the cancer epigenomic landscape.

Although this study focused on DNAm-linked isoform switching in cancer, some of the trends we identified are consistent with previous findings in normal cells. For example, we found DNAm near the 5’ end of a gene was primarily negatively correlated with the use of nearby TSSs, which is consistent with its previously documented repression of promoter activity [13, 27, 28]. We also noted that a few TSS showed positive correlations with methylation, which we predict this may occur when repression of one weakly defined TSS enables activation of another in close proximity. Such examples of proximal TSSs are strongly associated with CpG islands [29]. In addition, our findings shed light on a subject of debate in the literature regarding the role of intragenic DNAm in transcript elongation [30–32] vs wholesale transcriptional repression [33]. Except for regions around first exons, we found primarily positive correlations between gene body DNAm and isoform usage, supporting a role of DNAm in transcriptional elongation.

Some trends identified in this study have not been previously reported in normal cells—or in any study of DNAm and isoform switching. For example, we showed that DNAm that is negatively correlated with nearby TSSs shows enrichment for OSRs (Fig 1C). In addition, our data suggest that exonic DNAm occurring far from exon boundaries in alternative terminal exons, may promote the inclusion of those exons. These correlations suggest a potential blueprint for DNAm-regulated isoform activities worthy of further experimental elucidation while controlling for other isoform-regulating mechanisms such as aforementioned splicing factor alterations and recently reported regulation via miRNA binding at 3’UTRs [34].

In the past, the connection between DNAm and dysregulated isoform usage in cancer has been overlooked for two main reasons. First, the main functional role established for cancer-associated DNAm alterations is gene silencing via promoter CGI methylation. Thus, most cancer studies have focused on causal relationships between aberrant promoter hypermethylation and tumor suppressor gene silencing [35], paying less attention to the functional consequences of intragenic DNAm alterations. Second, the impact of DNAm alterations on the transcriptome is mostly studied at the gene rather than the isoform level. For example, to detect promoter DNAm-induced gene silencing and to study the impact of intragenic DNAm on gene transcription, gene-level expression data are used [36]. However, tumor samples with similar gene expression levels can display significant differences in isoform usage [4]. Our findings emphasize the need to conduct analyses at the isoform level in order to understand the impact of DNAm on the transcriptome.

Our study has several important limitations. First, the potential regulatory paradigms reported in this study (e.g., DNAm around the TSS is correlated with decreased isoform usage) may vary in a condition-specific manner. Our findings were based on majority rule, but we noted a few exceptions, including positive correlations between DNAm and nearby TSSs at some sites and negative correlations between DNAm and expression of isoform bodies at other sites. Second, our analysis was based on correlations between methylation at individual CpG sites and usage of individual isoforms. Whether (and how) DNAm at CpGs dispersed throughout a locus collectively regulates isoform usage is still unclear. Third, we did not directly investigate the role of DNAm in alternative splicing, although such alterations have been shown to contribute to cancer progression [7], and can be modulated by interactions between DNAm and proteins such as CTCF [15], MeCP2 [14] and HP1 [37]. Integrative analysis of these factors (using ChIP-Seq datasets, for example) coupled with exon-level expression data may provide insights into a mechanistic link between DNAm alterations and deleterious alternative splicing in cancer. Fourth, our analysis relied on Ensembl gene annotation. Thus, we may have missed links between DNAm and aberrantly expressed isoforms in cancer that have not yet been annotated. Finally, DNAm detected in this study could not distinguish its most abundant form, 5-mC, from other variants such as 5-hmC, 5-fC, and 5-caC, which might have different roles in isoform regulation.

In conclusion, this comprehensive analysis highlights the potential functional role of DNAm in dysregulating isoform usage in cancer. These findings provide new insights into the mechanistic links connecting DNAm and cancer, as well as the rules defining DNAm-isoform interactions. Experimentally validating the rules defining correlated DNAm-isoform expression in the future could give us more comprehensive understanding of cancer biology. Most importantly, given recent advances in targeted DNAm editing [38], if researchers are able to identify DNAm-induced isoform switching that drives cancer progression, the findings could launch a new field focused on epigenetic therapy.

## Methods

We analyzed samples for which both isoform expression data and DNA methylation data were available. These samples represented 18 cancer types: bladder urothelial carcinoma (BLCA), breast invasive carcinoma (BRCA), colon adenocarcinoma (COAD), glioblastoma multiforme (GBM), head and neck squamous cell carcinoma (HNSC), kidney renal clear cell carcinoma (KIRC), kidney renal papillary cell carcinoma (KIRP), liver hepatocellular carcinoma (LIHC), lung adenocarcinoma (LUAD), lung squamous cell carcinoma (LUSC), pancreatic adenocarcinoma (PAAD), prostate adenocarcinoma (PRAD), rectum adenocarcinoma (READ), skin cutaneous melanoma (SKCM), stomach adenocarcinoma (STAD), testicular germ cell tumors (TGCT), thyroid carcinoma (THCA), and uterine corpus endometrial carcinoma (UCEC). Out of 18 cancer types, 11 types were used to conduct most analyses in this study. The number of tumors and controls (tissue samples adjacent to tumors or from healthy donors) for each of the 11 cancer types is listed in Table 1.

### Data preprocessing

TCGA level 3 DNA methylation array-based data (Illumina Infinium HumanMethylation450 BeadChip array) were downloaded from the UCSC Cancer Genomics Browser (https://genome-cancer.ucsc.edu) on October 26, 2015. DNA methylation levels were measured with β values. We normalized β values for type I and II probes using the β mixture quantile method [39]. The following types of probes were removed from the analysis: (*i*) probes on the X and Y chromosomes, (*ii*) cross-reactive probes [40], (*iii*) probes near single nucleotide polymorphisms, and (*iv*) probes with missing rates ≥ 90% across all samples for a given cancer type. A final set of 314,421 probes was analyzed for each cancer type.

TCGA level-3 gene expression data measured by TPM-normalized RNA-seq (Illumina HiSeq) counts were downloaded from Google cloud pilot RNA-Sequencing for the Cancer Cell Line Encyclopedia and TCGA [41] (https://osf.io/gqrz9/) on November, 2016. Lowly expressed transcripts (median TPM ≤ 0) were removed. Genes with any of following conditions were also removed: (i) with only one isoform, (ii) on the sex chromosomes, or (iii) with no methylation probe in the intragenic regions (defined as the gene segment plus 2kb up/downstream based on Ensembl gene annotation).

TCGA tumor subtype classification was obtained from TCGA clinical data, which was downloaded from the UCSC Cancer Genomics Browser (https://genome-cancer.ucsc.edu) and TCGA publication website (https://cancergenome.nih.gov/publications).

### Statistical significance of isoform-probe correlation

The empirical false discovery rate of isoform-probe correlation was estimated by permuting sample labels for each isoform-probe pair.

### Batch effect analysis

We were able to obtain batch IDs for 14 cancer types and checked if isoform-probe correlation was driven by batch effects. Samples without batch IDs and methylation probes with NA values were removed. We first computed dispersion separability criterion (DSC) to quantify batch effects in the 14 types [42]. Then, batch effects were removed using the R package limma and the statistical significance of isoform-probe correlation was re-evaluated the same as above for the 14 types.

### Analyses for correlations between DNAm and isoform usage within or around the first, middle, and terminal exons

Each analysis only included relevant isoform-probe pairs depending on the probe site location in the exon-intron structure of the paired isoform. For example, the analysis for DNAm-isoform correlation around the first exon only considered isoform-probe pairs whose probes were located in or around the first exon of the paired isoforms. Thus, a probe paired with multiple isoforms could be counted multiple times in one or more analyses. For example, using this approach, a probe *p* could be located in the first exon of isoform *A* but in the second intron of isoform *B* in two distinct isoform-probe pairs (*A*-*p* and *B*-*p*). When we analyzed probes inside or around the first exon, the pair *A*-*p* would be included in the analysis, but the pair *B*-*p* would not (S17 Fig).

In analyses of DNAm-isoform correlations around the first, middle, and terminal exons, counts of isoform-probe pairs were binned into regions near (<1kb) and far (≥1kb) from the exon boundaries if probes were inside the exon. For pairs in which probes were outside the exon, we then computed counts across 500 bp sliding windows, moving outward from the exon boundaries up to 2000 bp up/downstream, and shifting 100 bp at a time. In cases where flanking exons were within 2000 bp regions, we only included the counts up to the boundaries of flanking exons.

Enrichment of positive/negative correlations for a particular bin/window as computed using the odds ratio. The odds were computed between the number of positive and non-positive (or negative and non-negative) correlations. The odds ratio was computed between the ratio in that bin and the ratio in other bins in the same analysis. The odds ratio of positive/negative correlations for OSRs in a bin was computed using the positive/negative odds in the OSRs versus positive/negative odds of CGI and SS regions in that same bin. Statistical significance was evaluated using hypergeometric test.

### Functional analysis for DNAm-correlated isoform switching in breast cancer

The analysis was restricted to correlated isoform-probe pairs in BRCA. Statistical significance of isoform usage changes between any pair of tumor subtypes and normal samples was evaluated using Wilcoxon rank sum test followed by Benjamini-Hochberg false discovery rate correction. We loaded data for statistical significance of DNAm-correlated isoform usage changes and corresponding Ensembl isoform annotations into the R package IsoformSwitchAnalyzeR [4] for each pair of subtypes (as two conditions to be compared for isoform switching). The package identified isoform switches between two subtypes using default parameters. External tools were used to predict coding potential ("Coding-Potential Assessment",[43], protein domains (Pfam,[44], and signaling peptide (SignalP 4.0, [45] for each isoform analyzed.

### Enrichment analysis for DNAm-isoform correlated genes in biological pathways and the known set of oncogenes and tumor suppressor genes

We tested whether DNAm-isoform correlated genes were overrepresented in cancer-related genes annotated by Cosmic [25] and TSGene [26] using hypergeometric test. For pathway enrichment analysis, we tested whether DNAm-isoform correlated genes were overrepresented in pathways in curated databases including KEGG [46], Reactome [47], NCI-Nature Interaction Pathway Database [48], and Gene Ontology (i.e., molecular function / biological process / cellular component), and gene sets characterized by Pfam/InterPro domains [44] using Enrichr [49].

### Statistical significance of association between DNA methylation clusters and correlated isoform usage patterns in LIHC, LUAD, LUSC, STAD, and UCEC

In S11 to S15 Figs, because association between DNA methylation clusters and isoform usage patterns was not visually clear, we evaluated whether the association was statistically significant. We first identified 4 methylation clusters using hierarchical clustering for each cancer type. Then we tested whether the isoform usage patterns were more similar within clusters than between cluster by computing the test statistic *T* as follows:

$$D_{w}=\sum_{c=1}^{4}\sum_{i=1}^{n_{c}}{\left(x_{i,c}-\mu_{c}\right)}^{T}(x_{i,c}-\mu_{c})$$

$$D_{b}=\sum_{c=1}^{4}n_{c}{\left(\mu_{c}-\mu \right)}^{T}(\mu_{c}-\mu )$$

$$T=D_{b}/D_{w}$$

where *x*_*i*,*c*_ is the isoform usage vector for sample *i* in cluster *c*, *μ_c_* is the mean isoform usage for cluster *c*, *n_c_* is the number of samples in cluster *c*, and *μ* is the mean isoform usage across all samples. Conceptually, *T* quantifies the ratio of between-cluster distance and within-cluster distance. *T* would be large if within-cluster isoform usages were similar compared to between-cluster isoform usages. We evaluated the statistical significance of the observed *T* using the null distribution of *T* constructed by permuting clustering labels 1,000 times.

## Supporting information

S1 Fig Significantly correlated isoform-DNA methylation probe pairs (empirical false discovery rate < 0.1) were observed in 16 out of 18 cancer types (not COAD and GBM), and significantly correlated isoform-probe pairs with a correlation > 0.3 were observed in 11 cancer types. (TIFF)

S2 Fig Significantly correlated isoform-DNA methylation probe pairs (empirical false discovery rate < 0.1) were observed in all 18 cancer types using Spearman correlation. (TIFF)

S3 Fig Significantly correlated isoform-DNA methylation probe pairs were not affected by batch effects in 14 cancer types where batch IDs were available. (A) Dispersion separability criterion (DSC) < 0.5 in each cancer type suggested batch effects were not very strong [42]. (B) Significant correlated isoform-DNA methylation probe pairs were still observed after removing batch effects.
(TIFF)

S4 Fig Positive correlations between DNA methylation and isoform use were enriched in isoform bodies (defined as regions downstream of the first exons) relative to isoform promoters (defined as the first exons and regions upstream of the TSS). The odds were computed between the ratio of positive versus non-positive correlations in isoform bodies and that in isoform promoters for each cancer type.
(TIFF)

S5 Fig DNA methylation in terminal exons that was located >1 kb away from both exon boundaries was positively correlated with isoform use, and these methylation sites disproportionately occurred in CPG islands (OR > 1). (TIFF)

S6 Fig Examples of DNAm correlated with inclusion of a long terminal exon and usage of a downstream alternative transcription termination site. (A) and (B) correspond to Fig 3C and 3D respectively, with functional domains also indicated.
(TIFF)

S7 Fig Link between DNAm patterns, isoform usage patterns, and previously identified subtypes in bladder cancer (BLCA). Figures were plotted in the same way as Fig 4A and 4B. Samples were clustered based on DNAm levels of isoform-correlated probes.
(TIFF)

S8 Fig Link between DNAm patterns, isoform usage patterns, and previously identified subtypes in head and neck cancer (HNSC). Figures were plotted in the same way as Fig 4A and 4B. Samples were clustered based on DNAm levels of isoform-correlated probes.
(TIFF)

S9 Fig Link between DNAm patterns, isoform usage patterns, and previously identified subtypes in prostate cancer (PRAD). Figures were plotted in the same way as Fig 4A and 4B. Samples were clustered based on DNAm levels of isoform-correlated probes.
(TIFF)

S10 Fig Link between DNAm patterns, isoform usage patterns, and previously identified subtypes in melanoma (SKCM). Figures were plotted in the same way as Fig 4A and 4B. Samples were clustered based on DNAm levels of isoform-correlated probes.
(TIFF)

S11 Fig Link between DNAm patterns, isoform usage patterns, and previously identified subtypes in liver cancer (LIHC). Figures were plotted in the same way as Fig 4A and 4B. Samples were clustered based on DNAm levels of isoform-correlated probes.
(TIFF)

S12 Fig Link between DNAm patterns, isoform usage patterns, and previously identified subtypes in lung adenocarcinoma (LUAD). Figures were plotted in the same way as Fig 4A and 4B. Samples were clustered based on DNAm levels of isoform-correlated probes.
(TIFF)

S13 Fig Link between DNAm patterns, isoform usage patterns, and previously identified subtypes in lung squamous cell carcinoma (LUSC). Figures were plotted in the same way as Fig 4A and 4B. Samples were clustered based on DNAm levels of isoform-correlated probes.
(TIFF)

S14 Fig Link between DNAm patterns, isoform usage patterns, and previously identified subtypes in stomach cancer (STAD). Figures were plotted in the same way as Fig 4A and 4B. Samples were clustered based on DNAm levels of isoform-correlated probes.
(TIFF)

S15 Fig Link between DNAm patterns, isoform usage patterns, and previously identified subtypes in uterine corpus endometrial carcinoma (UCEC). Figures were plotted in the same way as Fig 4A and 4B. Samples were clustered based on DNAm levels of isoform-correlated probes.
(TIFF)

S16 Fig Number of genes predicted to be functionally affected by DNAm-correlated isoform switching among breast cancer subtypes and normal samples. Each bar represents the number of genes affected by a particular type of functional change due to DNAm-correlated isoform switching from subtype A to subtype B, as predicted by IsoformSwitchAnalyzeR [4].
(TIFF)

S17 Fig A schematic plot illustrates how isoform-probe pairs were analyzed in this study. (TIFF)

S1 Table Results of enrichment test of cancer-related genes in DNAm-isoform correlated gene sets. DNAm-isoform correlated genes that were shared across at least *n* cancer types (where *n* = 2 to 11) were assessed in datasets from Cosmic [25] and TSGene, a tumor suppressor gene database [26] using the hypergeometric test.
(DOCX)

S2 Table Results of enrichment test of biological pathways and gene sets in DNAm-isoform correlated genes. Among 1,222 DNA methylation–isoform correlated genes shared across at least 6 out of 11 cancer types, biological pathways and gene sets from seven curated databases were enriched, as determined using Enrichr [49].
(XLSX)
