# Supplementary figures and images for: Aberrant DNA methylation defines isoform usage in cancer, with functional implications
Source: PLoS Comput Biol. 2019 Jul 22;15(7):e1007095. doi: 10.1371/journal.pcbi.1007095 (PMC6675117; doi:10.1371/journal.pcbi.1007095)

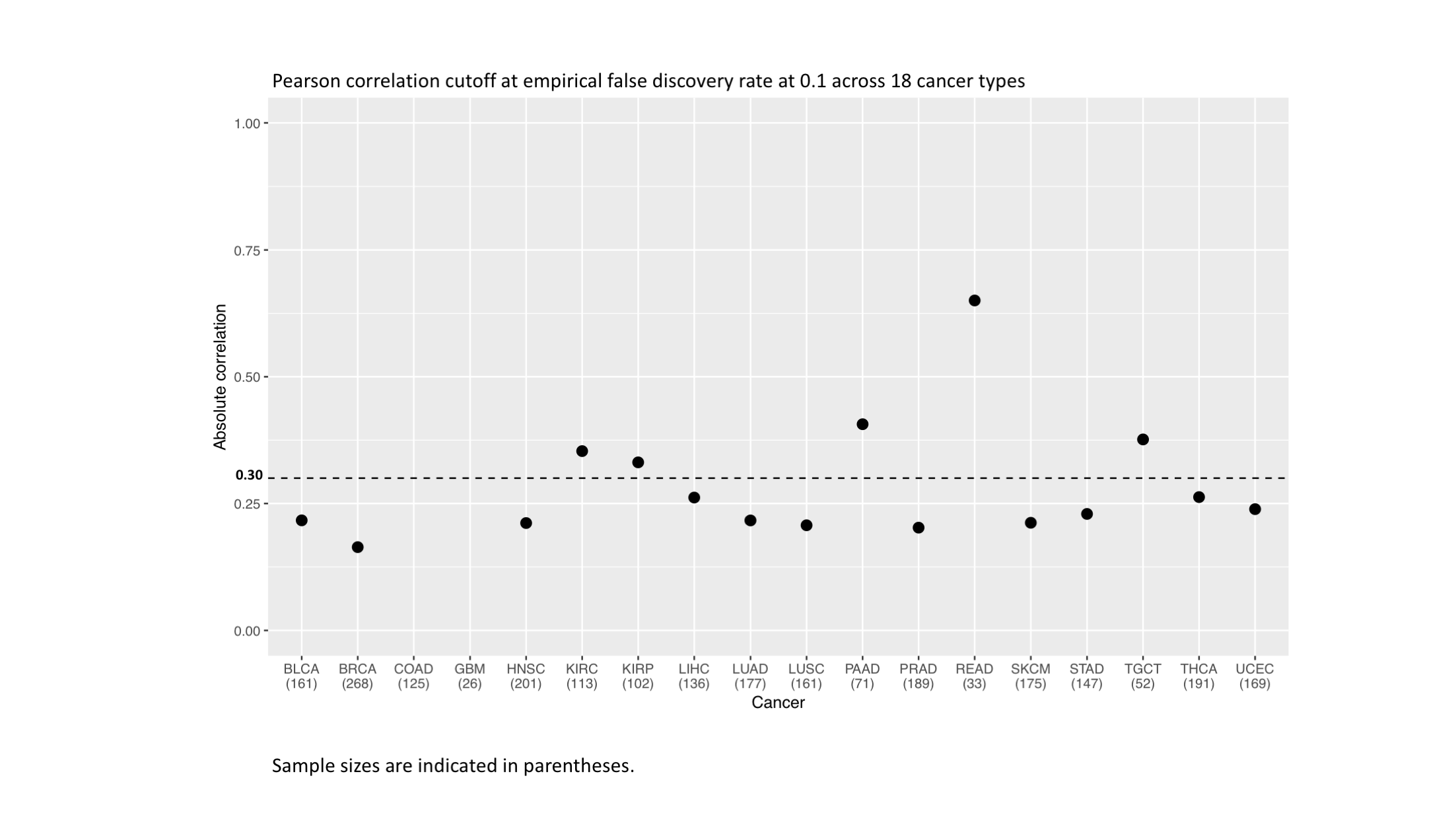

Supplement: S1 Fig — (TIFF) [file pcbi.1007095.s001.tiff]

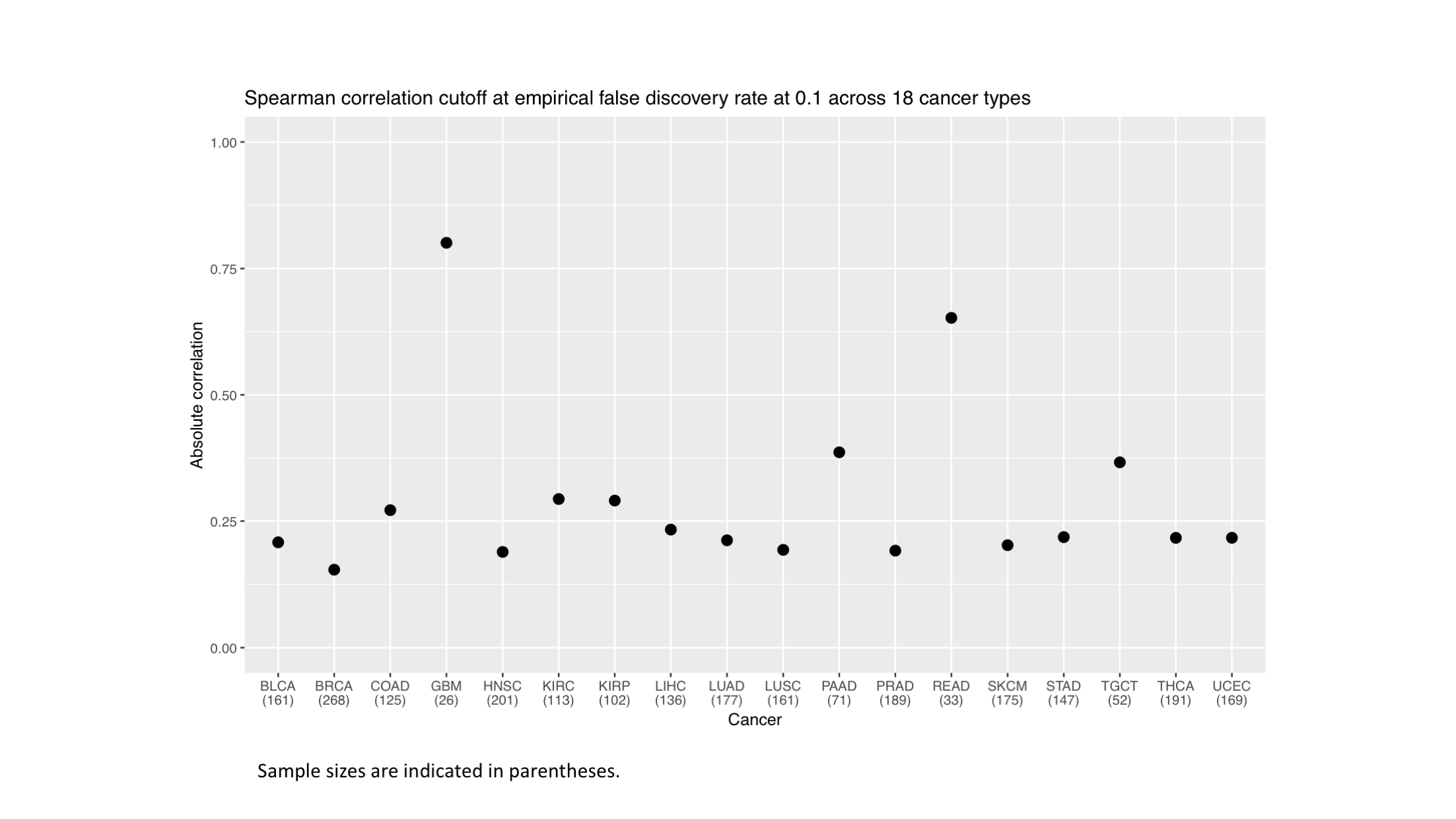

Supplement: S2 Fig — (TIFF) [file pcbi.1007095.s002.tiff]

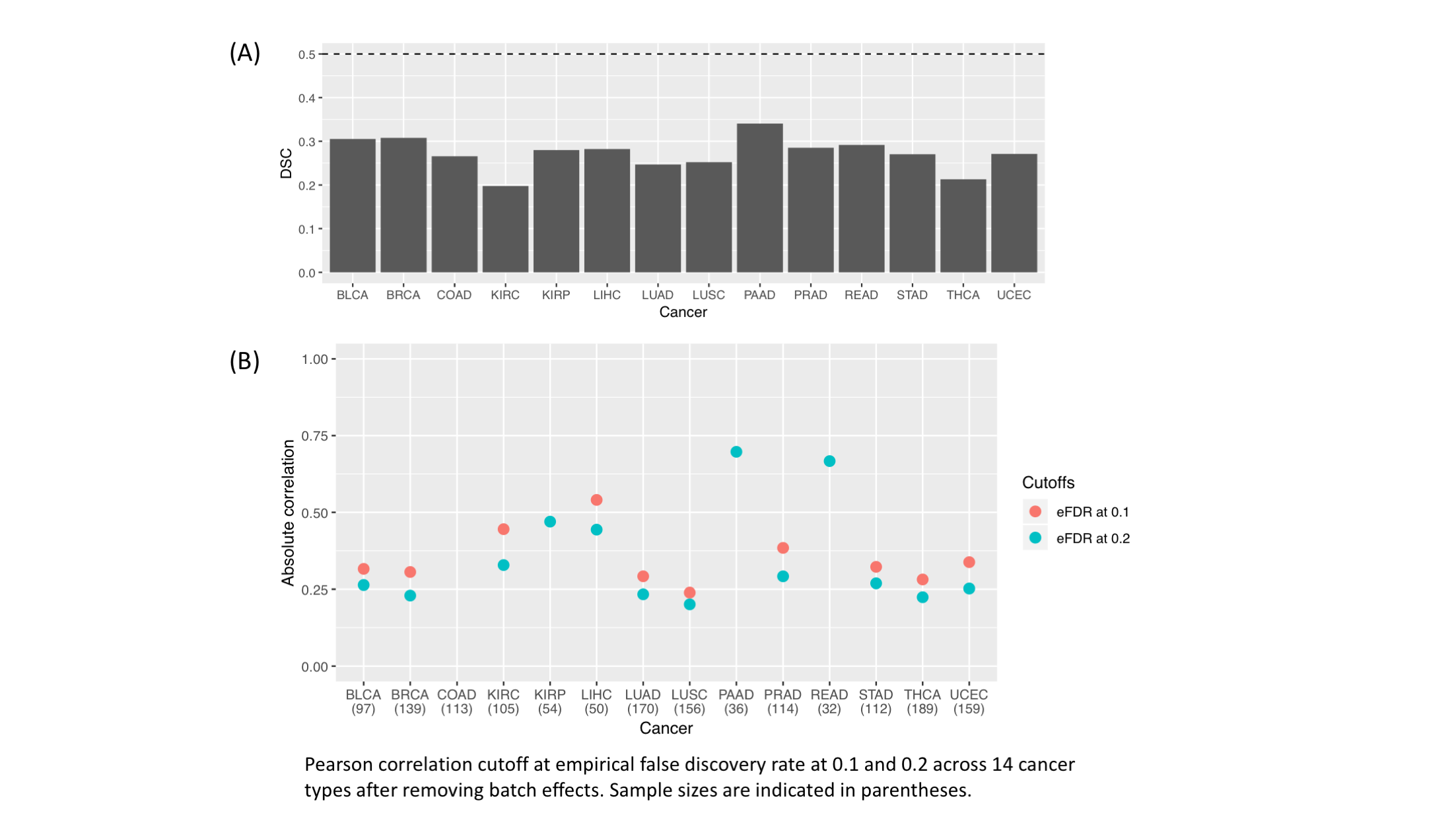

Supplement: S3 Fig — (A) Dispersion separability criterion (DSC) < 0.5 in each cancer type suggested batch effects were not very strong [42]. (B) Significant correlated isoform-DNA methylation probe pairs were still observed after removing batch effects. (TIFF) [file pcbi.1007095.s003.tiff]

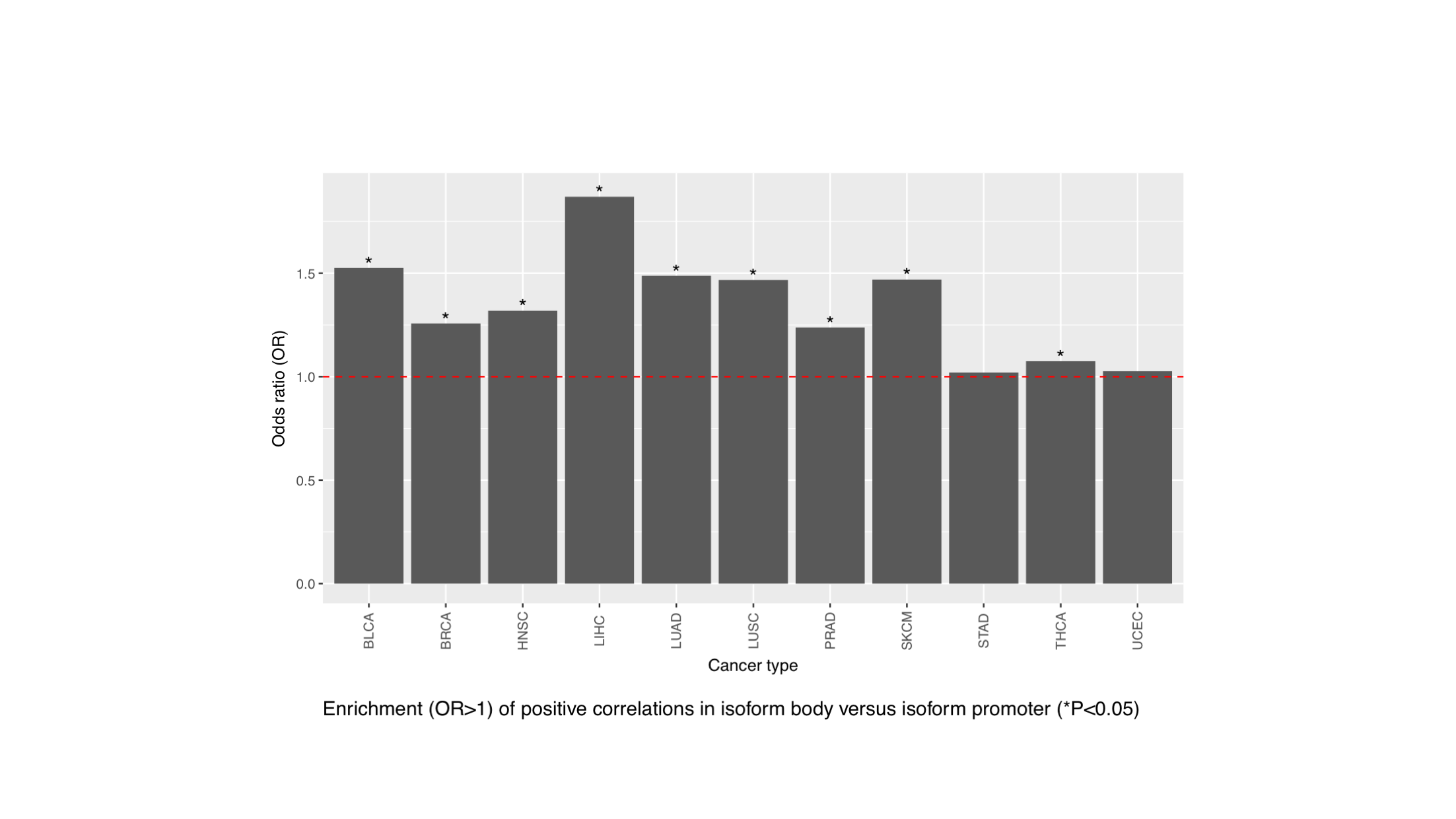

Supplement: S4 Fig — The odds were computed between the ratio of positive versus non-positive correlations in isoform bodies and that in isoform promoters for each cancer type. (TIFF) [file pcbi.1007095.s004.tiff]

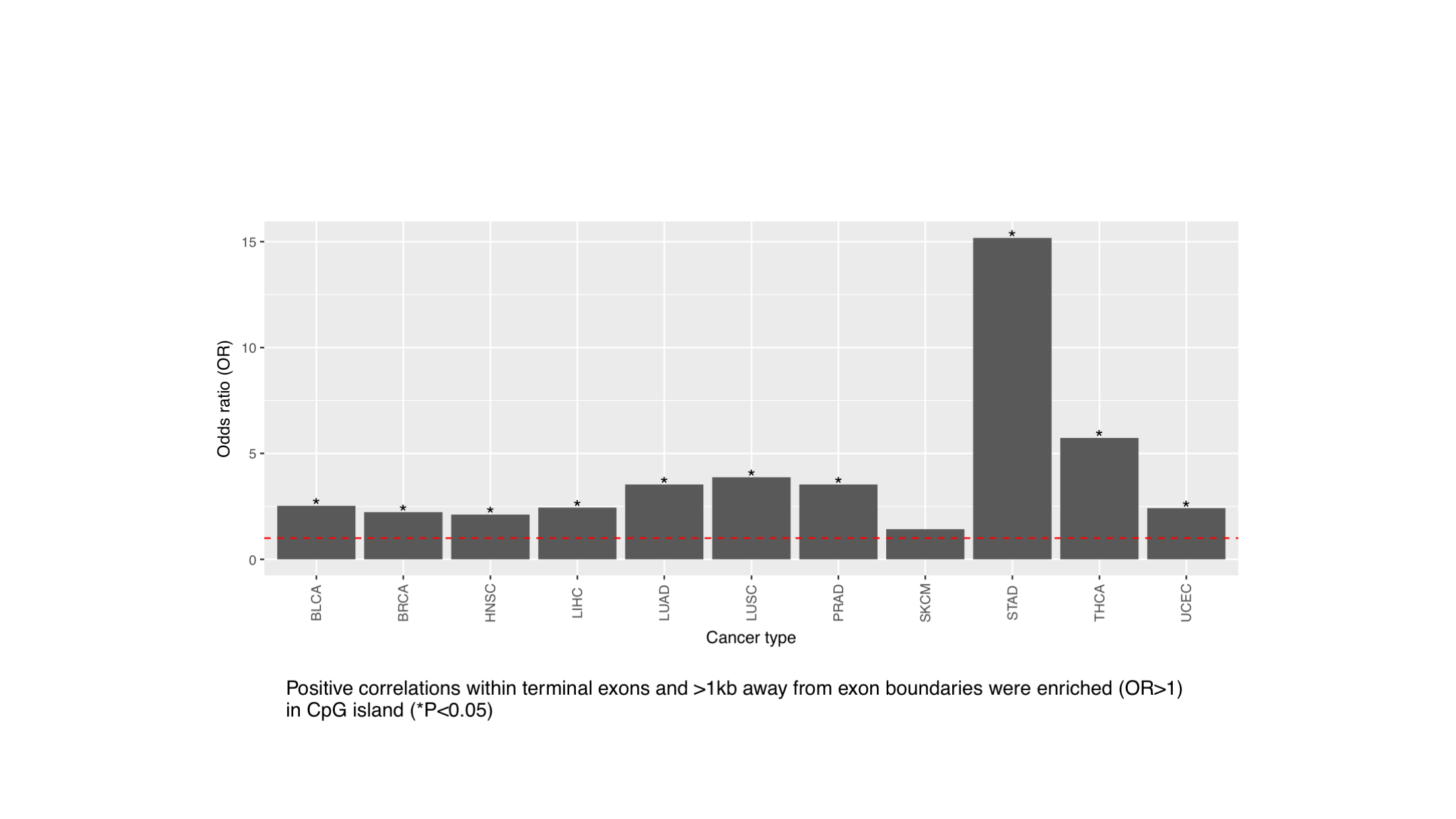

Supplement: S5 Fig — (TIFF) [file pcbi.1007095.s005.tiff]

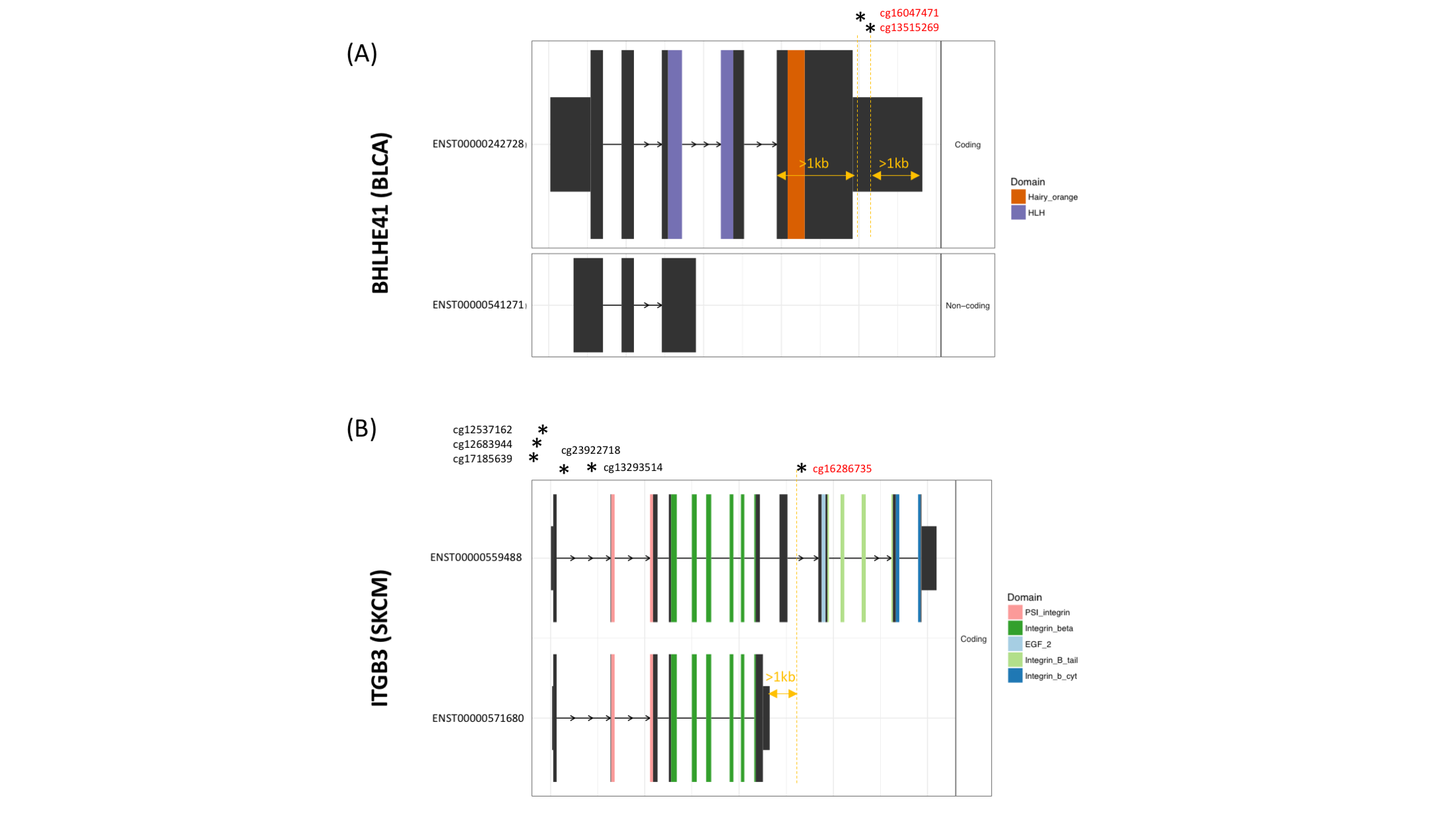

Supplement: S6 Fig — (A) and (B) correspond to Fig 3C and 3D respectively, with functional domains also indicated. (TIFF) [file pcbi.1007095.s006.tiff]

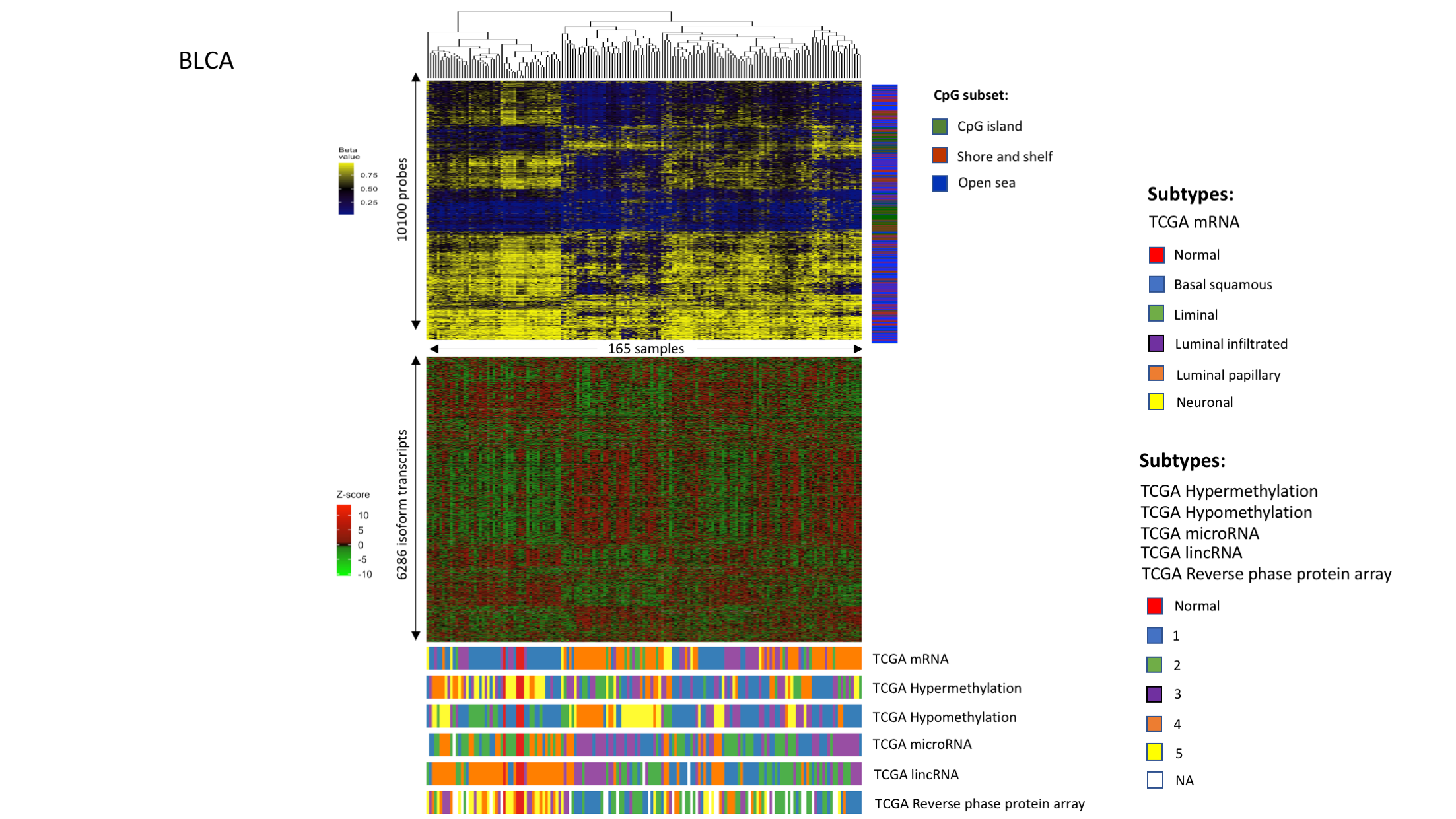

Supplement: S7 Fig — Figures were plotted in the same way as Fig 4A and 4B. Samples were clustered based on DNAm levels of isoform-correlated probes. (TIFF) [file pcbi.1007095.s007.tiff]

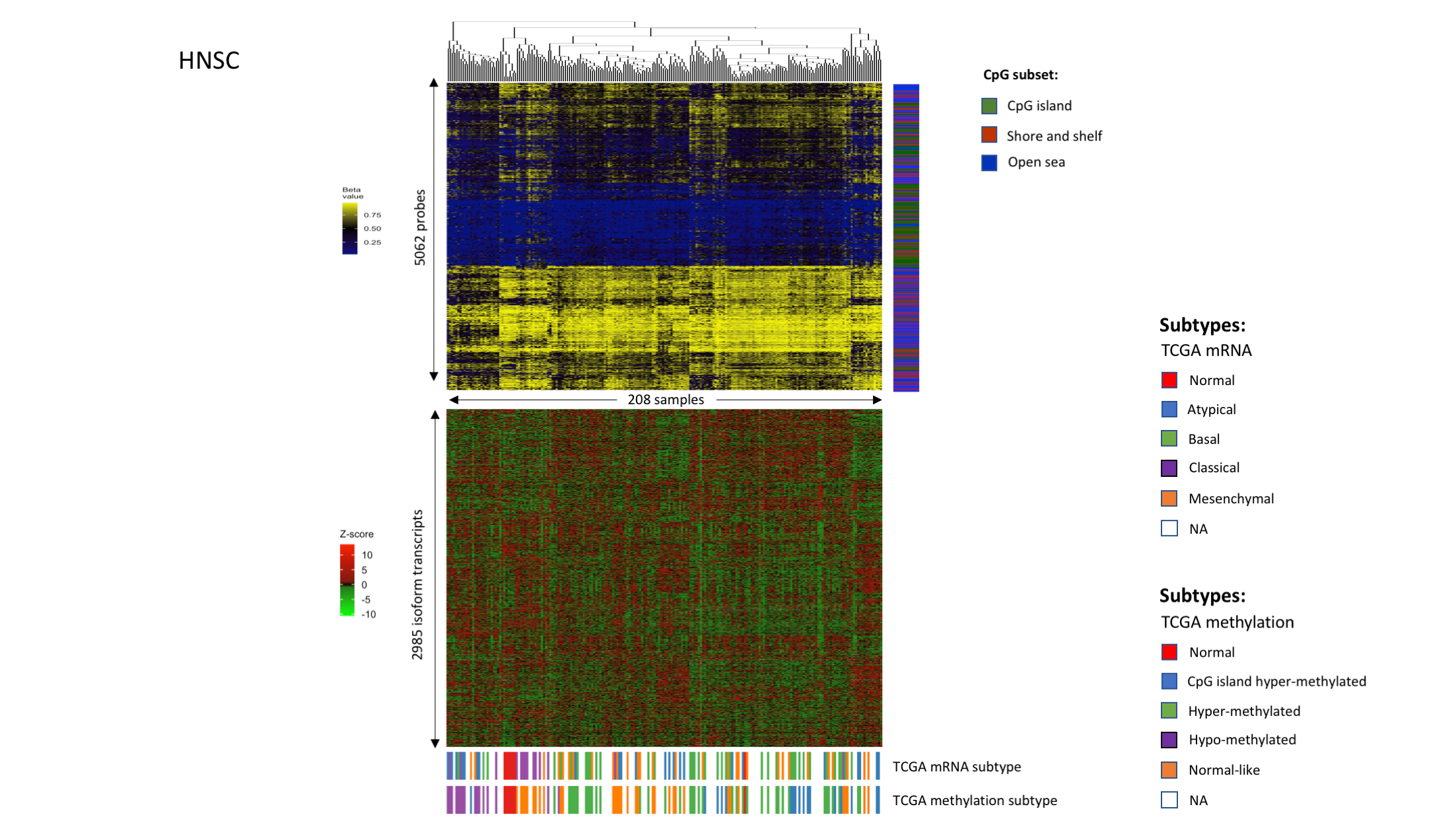

Supplement: S8 Fig — Figures were plotted in the same way as Fig 4A and 4B. Samples were clustered based on DNAm levels of isoform-correlated probes. (TIFF) [file pcbi.1007095.s008.tiff]

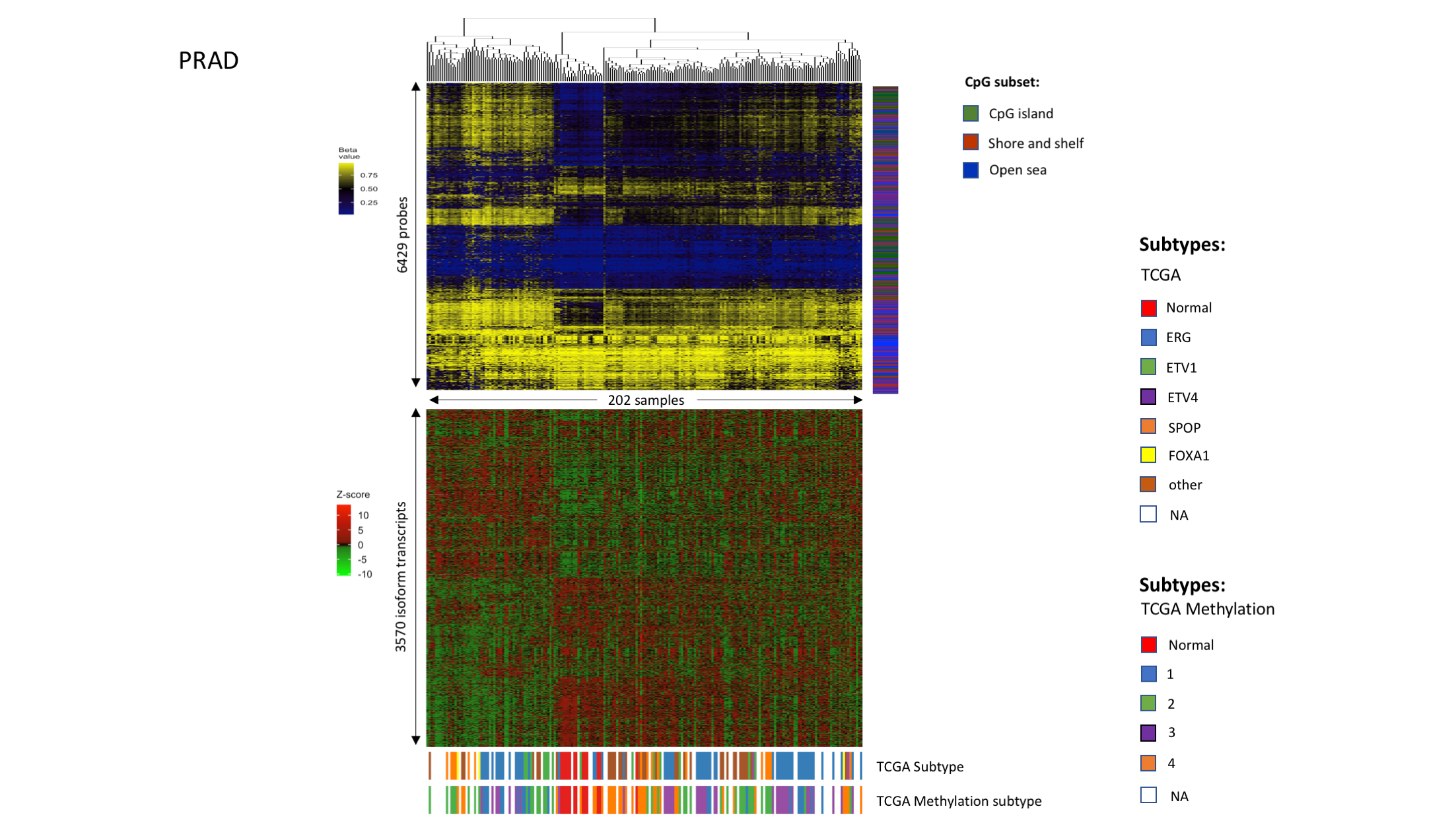

Supplement: S9 Fig — Figures were plotted in the same way as Fig 4A and 4B. Samples were clustered based on DNAm levels of isoform-correlated probes. (TIFF) [file pcbi.1007095.s009.tiff]

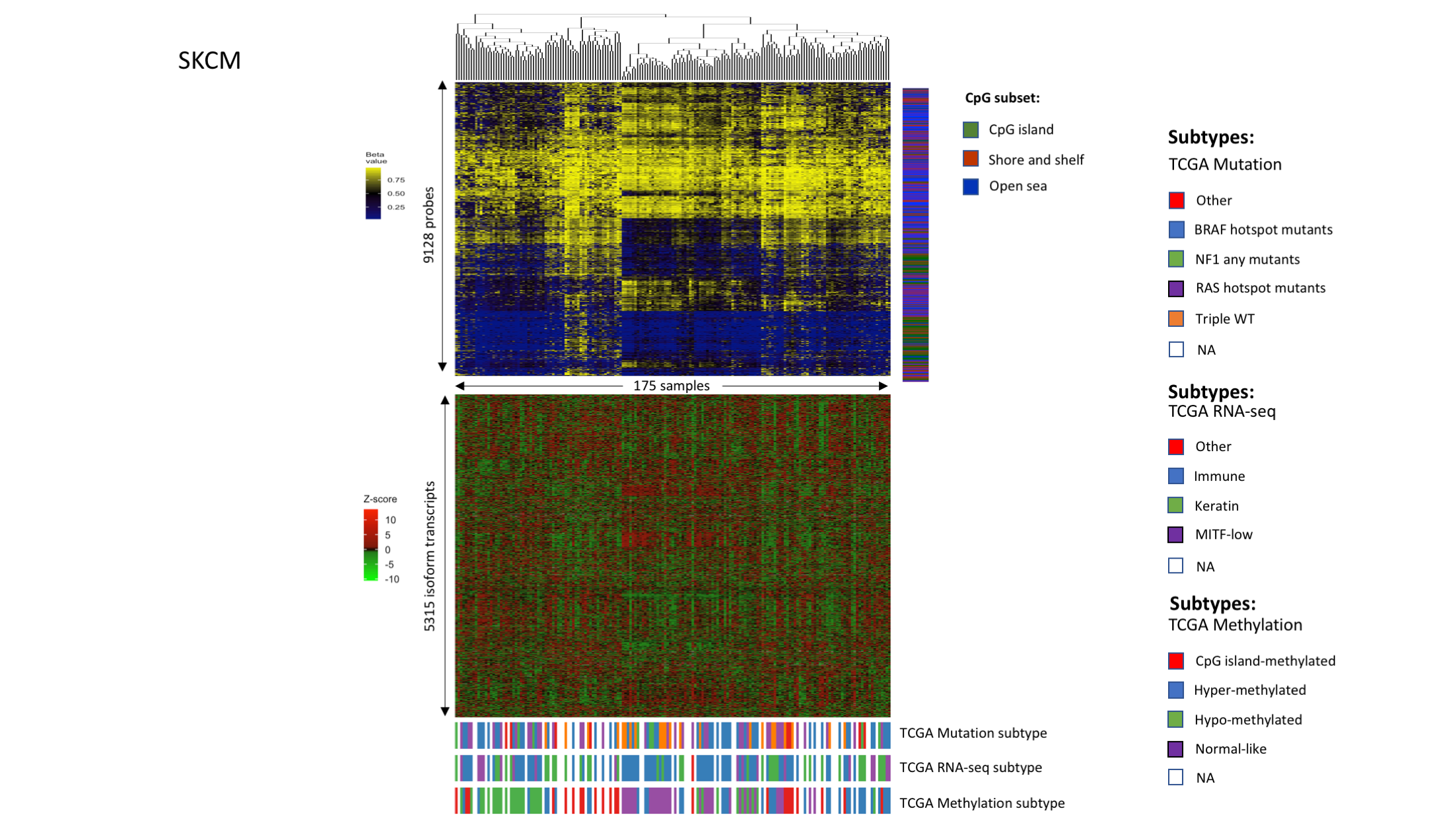

Supplement: S10 Fig — Figures were plotted in the same way as Fig 4A and 4B. Samples were clustered based on DNAm levels of isoform-correlated probes. (TIFF) [file pcbi.1007095.s010.tiff]

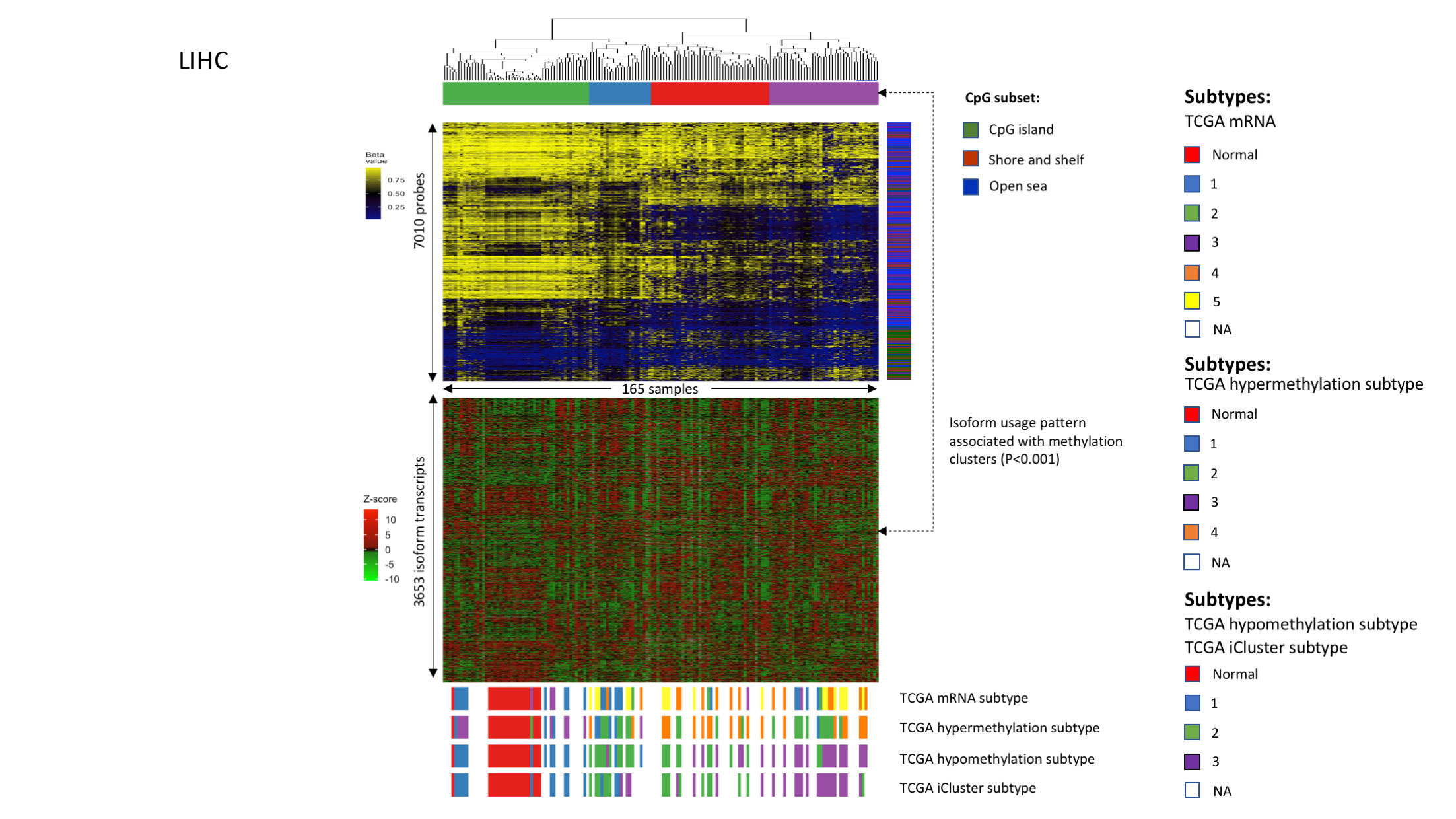

Supplement: S11 Fig — Figures were plotted in the same way as Fig 4A and 4B. Samples were clustered based on DNAm levels of isoform-correlated probes. (TIFF) [file pcbi.1007095.s011.tiff]

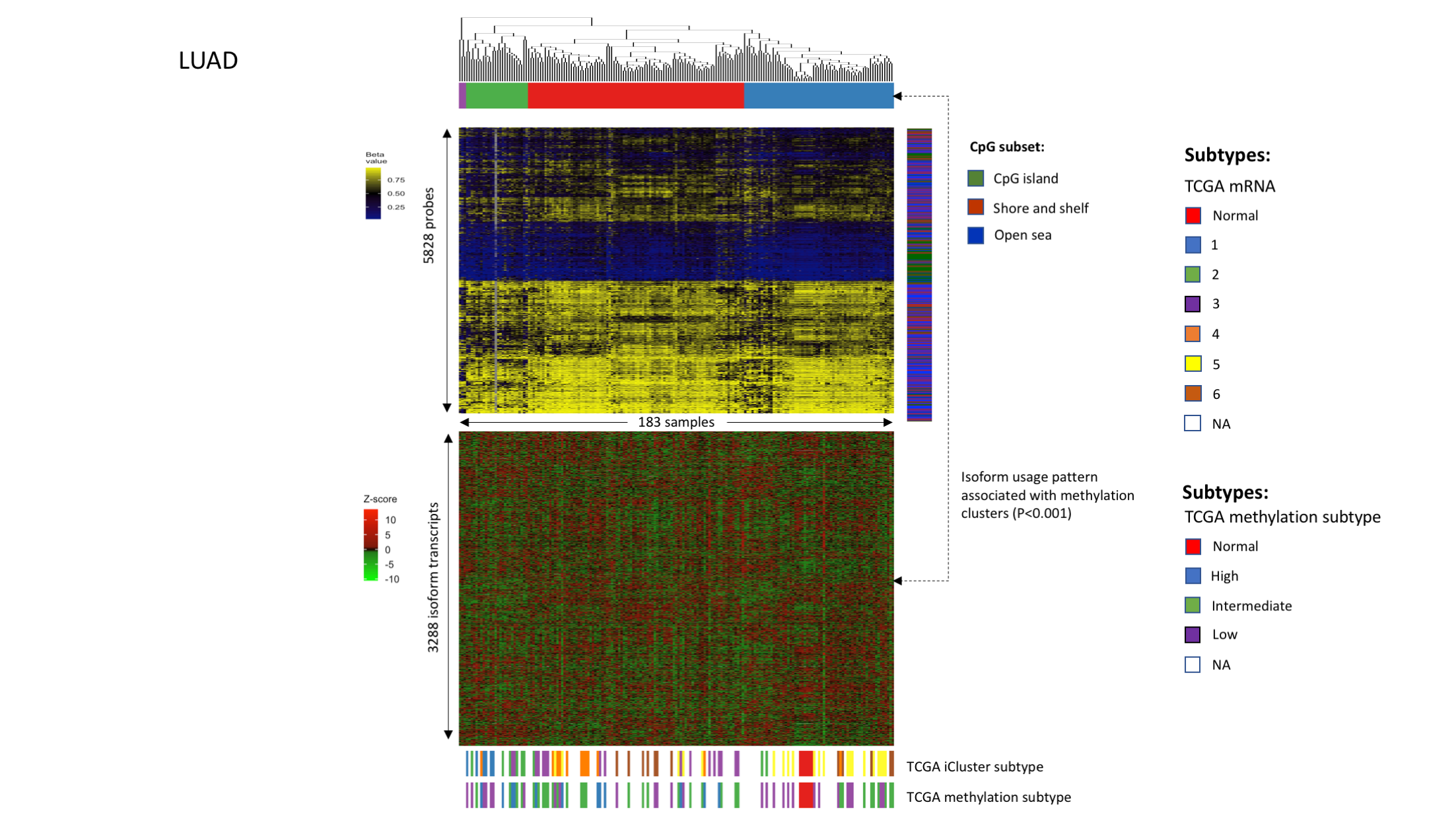

Supplement: S12 Fig — Figures were plotted in the same way as Fig 4A and 4B. Samples were clustered based on DNAm levels of isoform-correlated probes. (TIFF) [file pcbi.1007095.s012.tiff]

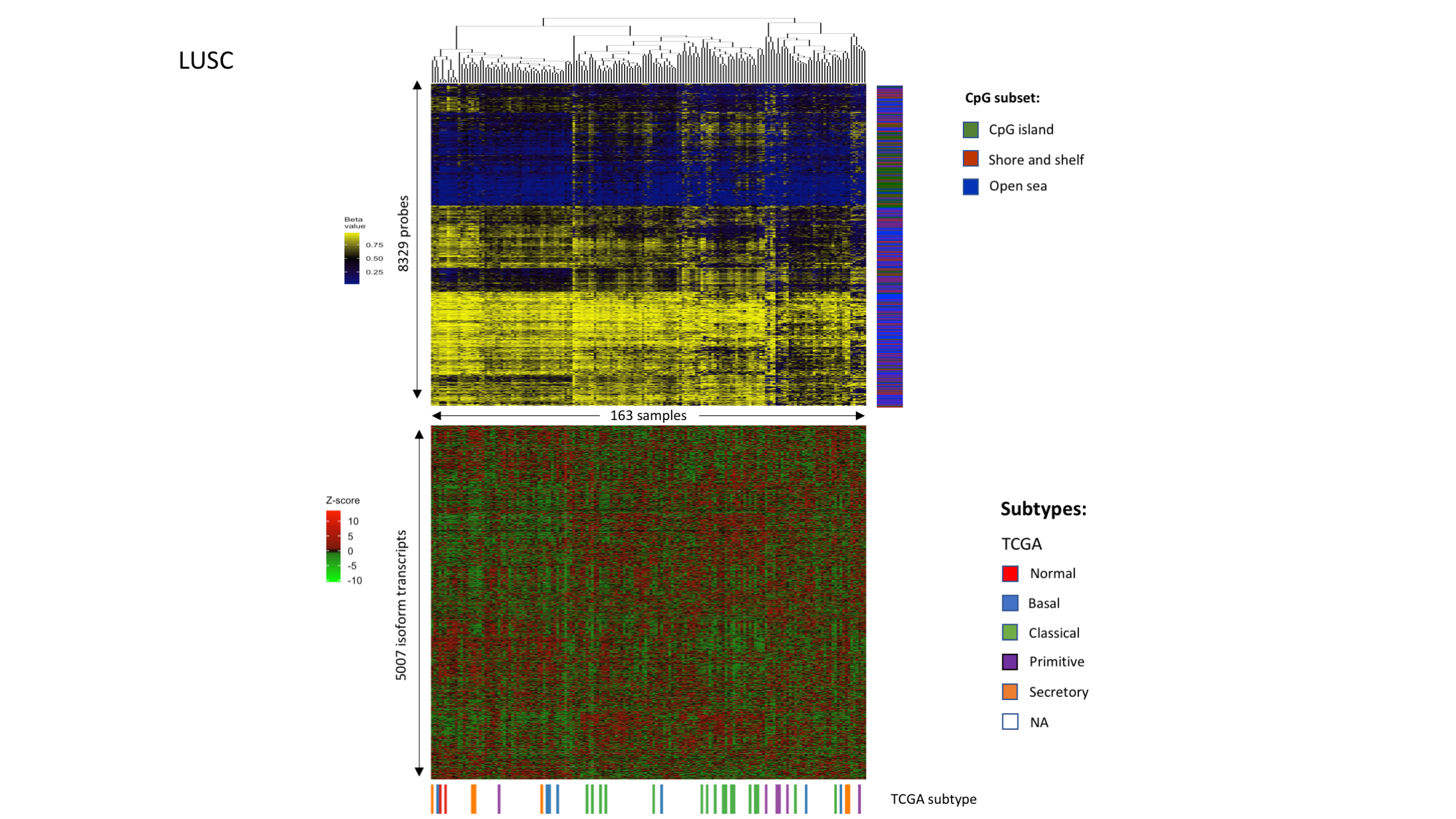

Supplement: S13 Fig — Figures were plotted in the same way as Fig 4A and 4B. Samples were clustered based on DNAm levels of isoform-correlated probes. (TIFF) [file pcbi.1007095.s013.tiff]

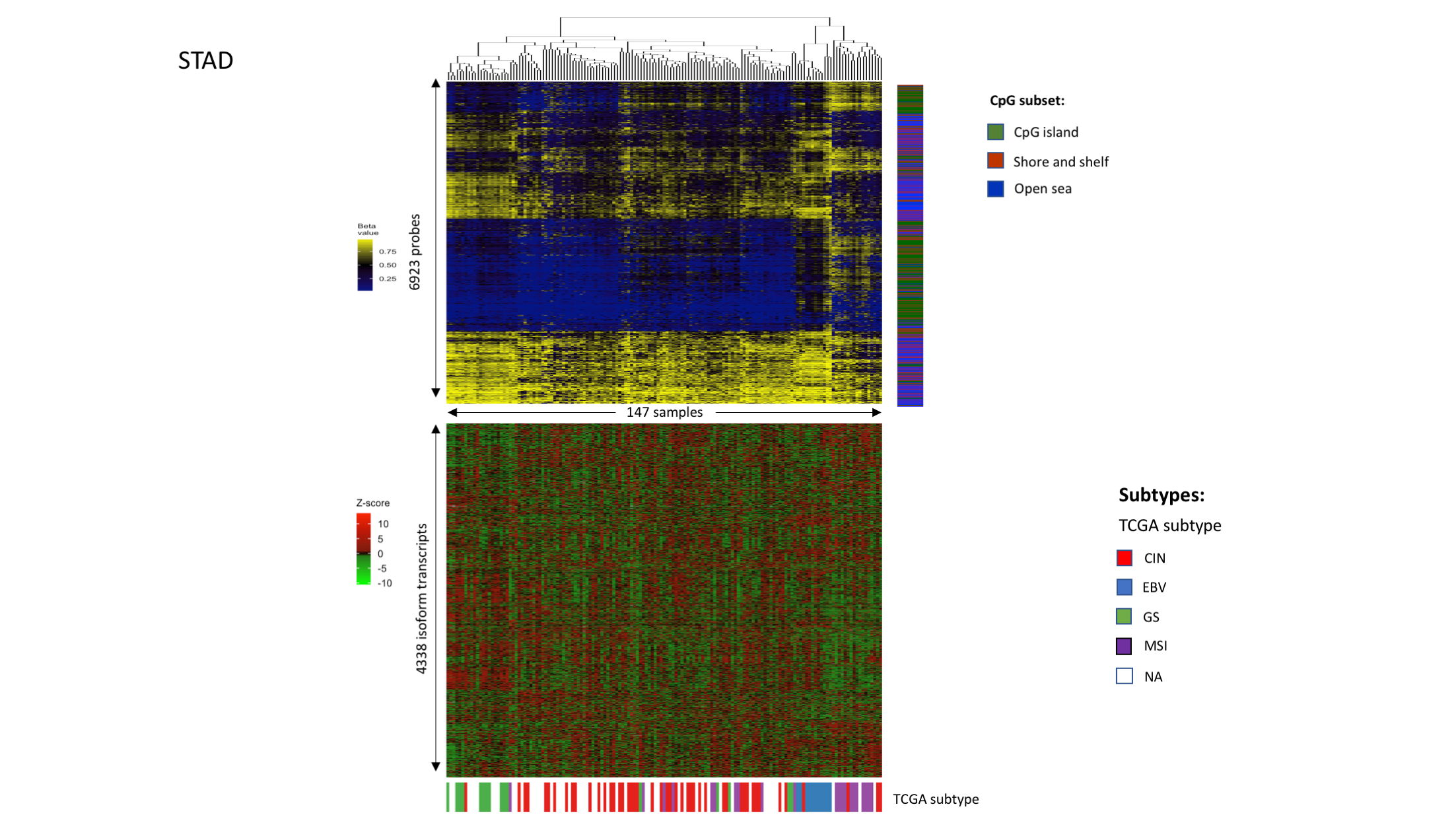

Supplement: S14 Fig — Figures were plotted in the same way as Fig 4A and 4B. Samples were clustered based on DNAm levels of isoform-correlated probes. (TIFF) [file pcbi.1007095.s014.tiff]

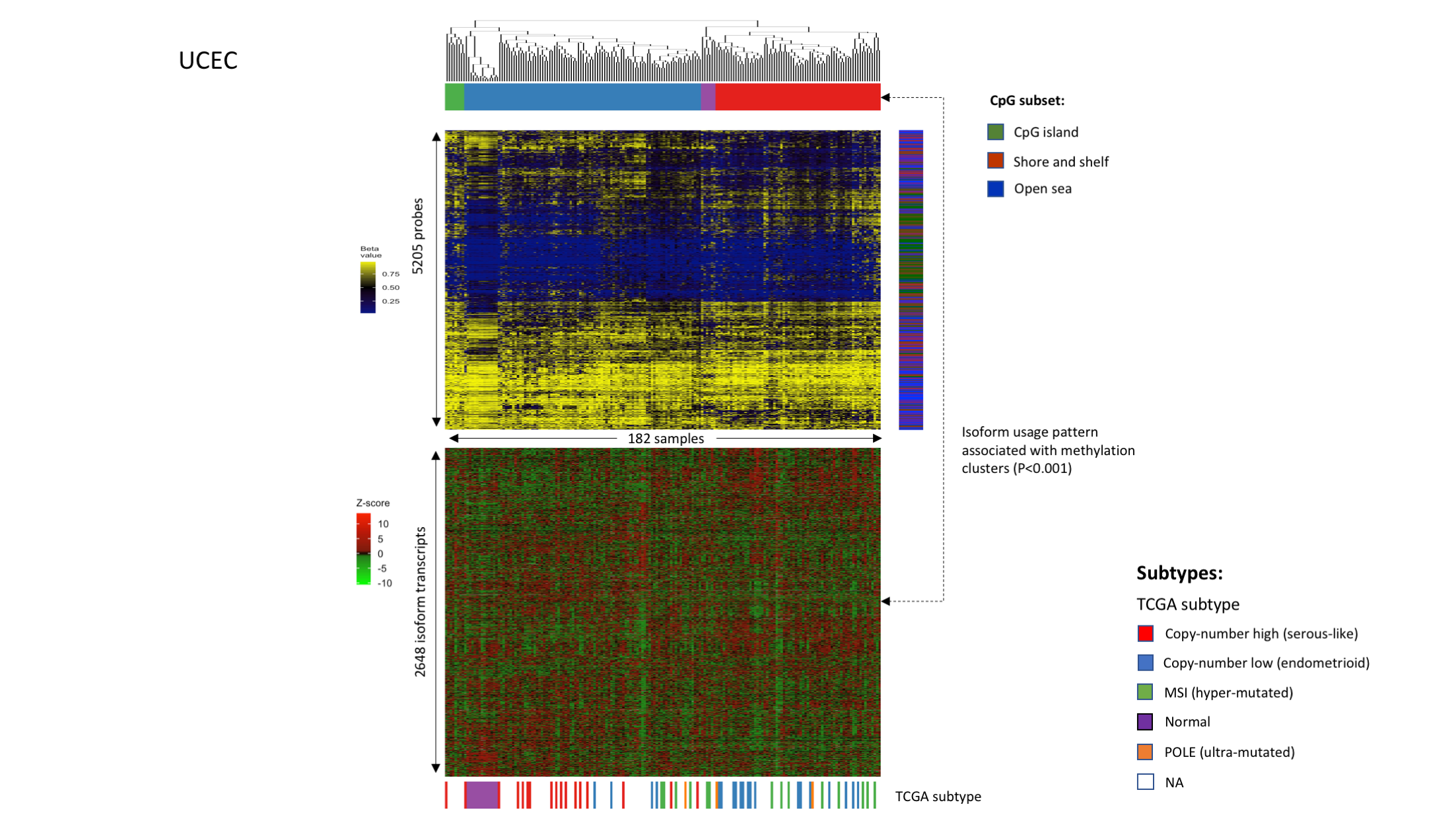

Supplement: S15 Fig — Figures were plotted in the same way as Fig 4A and 4B. Samples were clustered based on DNAm levels of isoform-correlated probes. (TIFF) [file pcbi.1007095.s015.tiff]

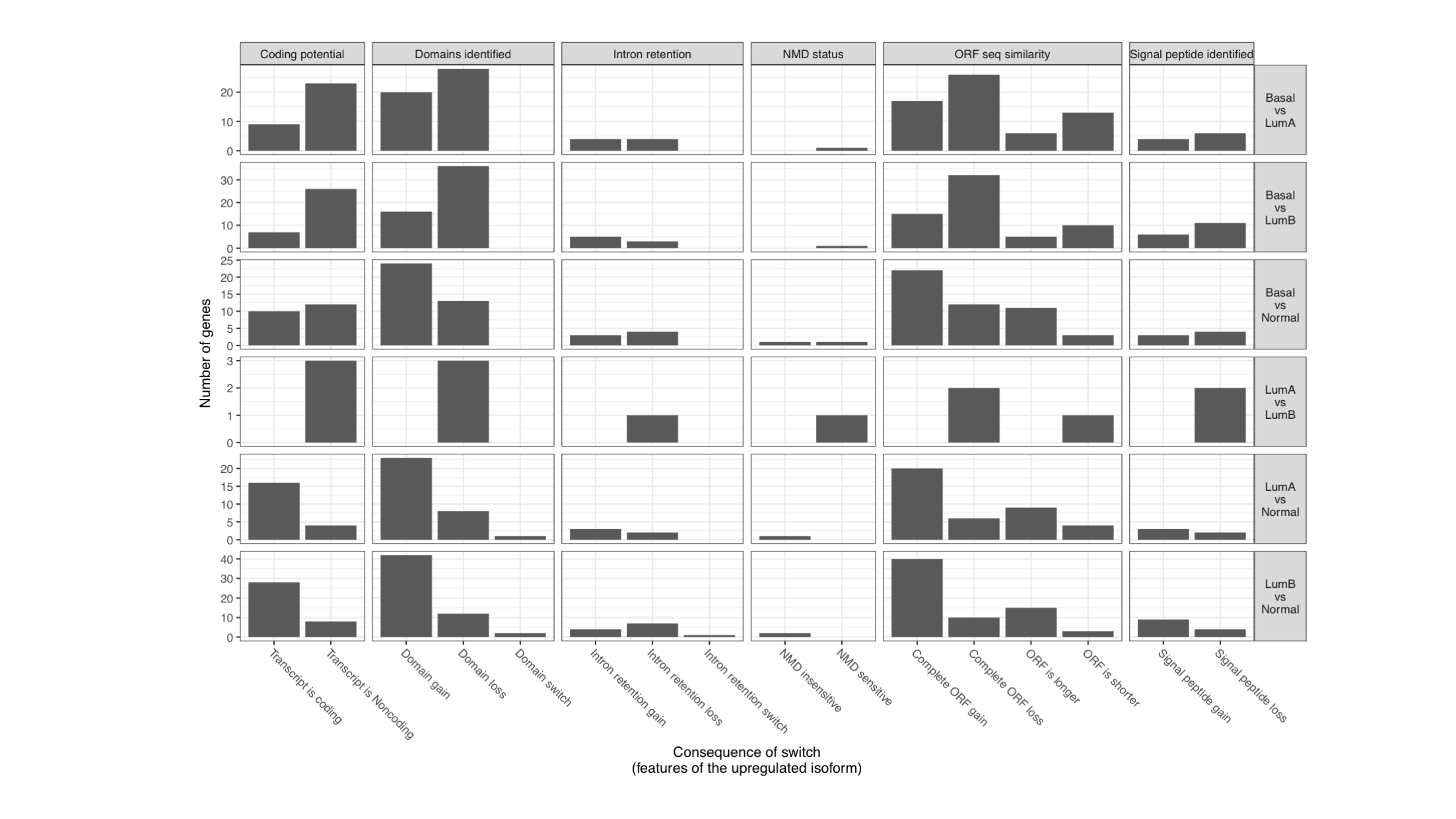

Supplement: S16 Fig — Each bar represents the number of genes affected by a particular type of functional change due to DNAm-correlated isoform switching from subtype A to subtype B, as predicted by IsoformSwitchAnalyzeR [4]. (TIFF) [file pcbi.1007095.s016.tiff]

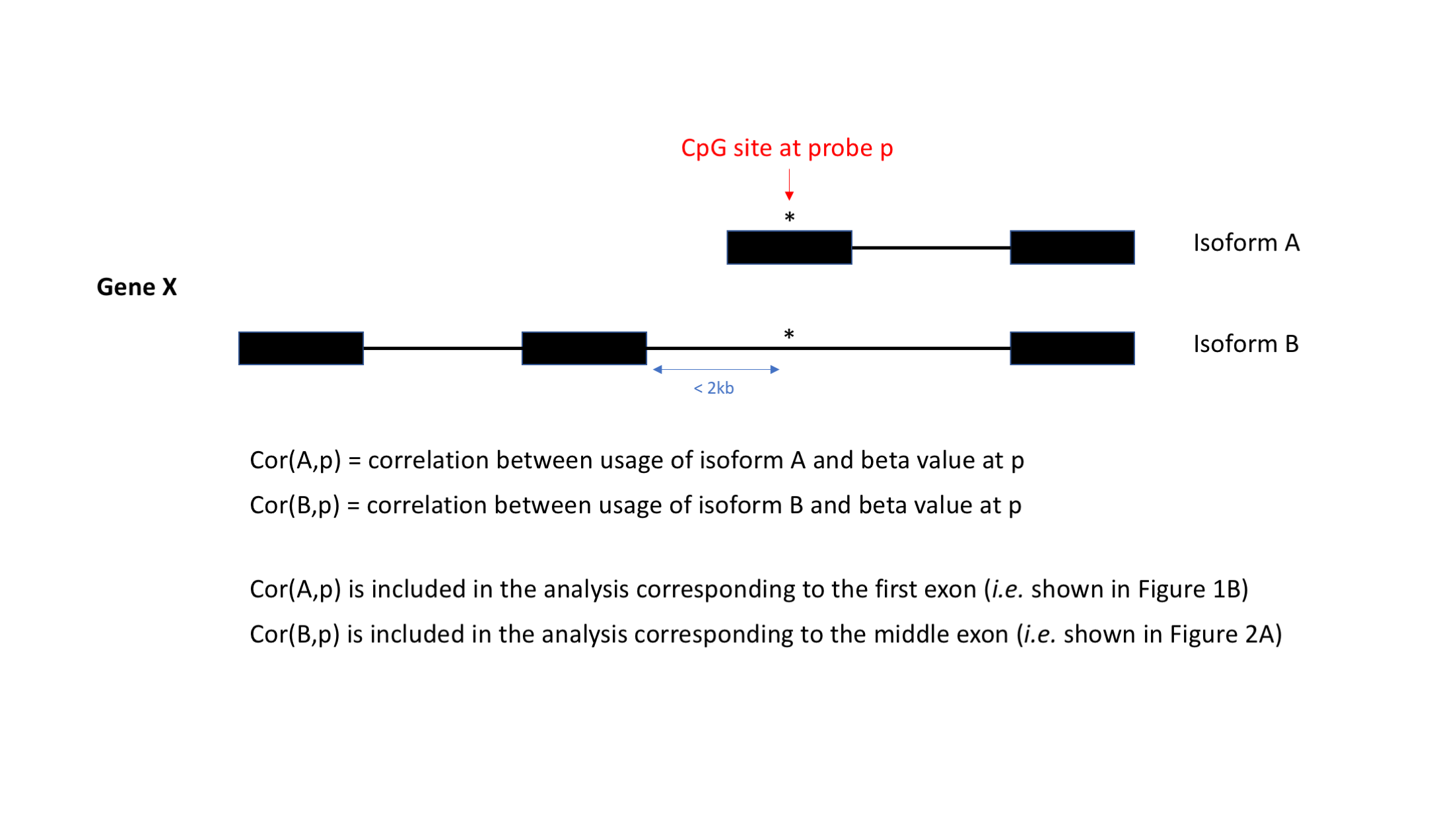

Supplement: S17 Fig — (TIFF) [file pcbi.1007095.s017.tiff]
