# Supplementary material for: Aberrant DNA methylation defines isoform usage in cancer, with functional implications
Source: PLoS Comput Biol. 2019 Jul 22;15(7):e1007095. doi: 10.1371/journal.pcbi.1007095 (PMC6675117; doi:10.1371/journal.pcbi.1007095)
Supplement: S1 Table — DNAm-isoform correlated genes that were shared across at least n cancer types (where n = 2 to 11) were assessed in datasets from Cosmic [25] and TSGene, a tumor suppressor gene database [26] using the hypergeometric test. (DOCX) [file pcbi.1007095.s018.docx]

| **# cancer types (n)** | **Total (K)** | **Cosmic/TSG (k)** | **Percentage (k/K)** | **P-value^*^** |
| --- | --- | --- | --- | --- |
| 11 | 57 | 9 | 16% | 7E-4 |
| 10 | 162 | 19 | 12% | 3.3E-4 |
| 9 | 339 | 28 | 8% | 7E-3 |
| 8 | 533 | 42 | 8% | 4E-3 |
| 7 | 847 | 69 | 8% | 1.1E-4 |
| 6 | 1222 | 106 | 9% | 9.4E-8 |
| 5 | 1698 | 151 | 9% | 2E-11 |
| 4 | 2364 | 218 | 9% | 2.3E-18 |
| 3 | 3237 | 272 | 8% | 1.3E-17 |
| 2 | 4674 | 374 | 8% | 7E-22 |

**Supplementary Table 1.**

^*^Hypergeometric test for a total number of 16,768 genes including 883 in COSMIC and TSG databases
